# Real-time denoising enables high-sensitivity fluorescence time-lapse imaging beyond the shot-noise limit

**DOI:** 10.1038/s41587-022-01450-8

**Published:** 2022-09-26

**Authors:** Xinyang Li, Yixin Li, Yiliang Zhou, Jiamin Wu, Zhifeng Zhao, Jiaqi Fan, Fei Deng, Zhaofa Wu, Guihua Xiao, Jing He, Yuanlong Zhang, Guoxun Zhang, Xiaowan Hu, Xingye Chen, Yi Zhang, Hui Qiao, Hao Xie, Yulong Li, Haoqian Wang, Lu Fang, Qionghai Dai

**Affiliations:** 1 Department of Automation, Tsinghua University, Beijing, China; 2 Tsinghua Shenzhen International Graduate School, Tsinghua University, Shenzhen, China; 3 Institute for Brain and Cognitive Sciences, Tsinghua University, Beijing, China; 4 Hangzhou Zhuoxi Institute of Brain and Intelligence, Hangzhou, China; 5 School of Information and Technology, Fudan University, Shanghai, China; 6 Beijing Key Laboratory of Multi-dimension & Multi-scale Computational Photography (MMCP), Tsinghua University, Beijing, China; 7 IDG/McGovern Institute for Brain Research, Tsinghua University, Beijing, China; 8 Department of Electronic Engineering, Tsinghua University, Beijing, China; 9 State Key Laboratory of Membrane Biology, Peking University School of Life Sciences, Beijing, China; 10 PKU-IDG/McGovern Institute for Brain Research, Beijing, China

**Keywords:** Fluorescence imaging, Microscopy, Image processing, Software

## Abstract

A fundamental challenge in fluorescence microscopy is the photon shot noise arising from the inevitable stochasticity of photon detection. Noise increases measurement uncertainty and limits imaging resolution, speed and sensitivity. To achieve high-sensitivity fluorescence imaging beyond the shot-noise limit, we present DeepCAD-RT, a self-supervised deep learning method for real-time noise suppression. Based on our previous framework DeepCAD, we reduced the number of network parameters by 94%, memory consumption by 27-fold and processing time by a factor of 20, allowing real-time processing on a two-photon microscope. A high imaging signal-to-noise ratio can be acquired with tenfold fewer photons than in standard imaging approaches. We demonstrate the utility of DeepCAD-RT in a series of photon-limited experiments, including in vivo calcium imaging of mice, zebrafish larva and fruit flies, recording of three-dimensional (3D) migration of neutrophils after acute brain injury and imaging of 3D dynamics of cortical ATP release. DeepCAD-RT will facilitate the morphological and functional interrogation of biological dynamics with a minimal photon budget.

## Abstract (web summary)

DeepCAD-RT denoises fluorescence time-lapse images in real time.

## Main

The proper functioning of living organisms relies on a series of spatiotemporally orchestrated cellular and subcellular activities. Observing and recording these phenomena are considered to be the first step toward understanding them. Fluorescence microscopy, combined with the growing palette of fluorescent indicators, provides biologists with a practical tool capable of good molecular specificity and high spatiotemporal resolution. Recent advances in fluorescence imaging have brought us insights into various previously inaccessible processes, ranging from nanoscale organelle interactions^1–3^ to pan-cell footprints during embryo development^4–6^ and whole-brain neuronal dynamics synchronized with certain behaviors^7–10^.

Among the challenges of fluorescence microscopy, poor imaging signal-to-noise ratio (SNR) caused by limited photon budget stands in the central position. The causes of this photon-limited challenge are manifold. First, the low photon yield of fluorescent indicators and their low concentration in labeled cells result in a lack of photons at the source^11^. Second, although using higher excitation power is a straightforward way to increase fluorescent photons, living systems are too fragile to tolerate high excitation dosage. Extensive experiments have shown that illumination-induced photobleaching, phototoxicity and tissue heating will disturb crucial cellular processes, including cell proliferation, migration, vesicle release, neuronal firing and so on^12–19^. Third, recording fast biological processes necessitates high imaging speed, and short dwell time further exacerbates the shortage of photons. Fourth, the quantum nature of photons makes the stochasticity (shot noise) of optical measurements inevitable^20,21^. The intensity detected by photoelectric sensors follows a Poisson distribution parameterized with the exact photon count^22^. In fluorescence imaging, detection noise dominated by photon shot noise aggravates the measurement uncertainty and obstructs the visualization of underlying structures, potentially altering morphological and functional interpretations that follow. To capture enough photons for satisfactory imaging sensitivity, researchers have to sacrifice imaging speed, resolution and even sample health^20,23^.

Comprehensive efforts have been invested to increase the photon budget of fluorescence microscopy, from designing high-performance fluorophores^11,24–26^ to upgrading the excitation and detection physics^20,27–29^ and developing data-driven denoising algorithms^23,30–33^. We previously developed DeepCAD, a deep self-supervised denoising method for calcium imaging data, which effectively suppresses the detection noise and improves imaging SNR more than tenfold without requiring any high-SNR observations^33^. A single low-SNR calcium imaging sequence can be directly used as the training data to train a denoising convolutional neural network (CNN).

Here, with advancements in methods and applications, we present DeepCAD-RT, a versatile self-supervised denoising method for fluorescence time-lapse imaging with real-time processing speed and improved performance. DeepCAD-RT inherits the self-supervised concept of splitting adjacent frames into inputs and corresponding targets to train a CNN^33^. By pruning redundant features inside the network architecture, we constructed a lightweight network and compressed the model parameters by 94%, which consequently reduced 85% processing time and 70% memory consumption. Meanwhile, we augmented the training data by 12-fold to alleviate the data dependency and make the method still tractable with a small amount of data. We show that such a strategy of combining model compression and data augmentation eliminates overfitting and makes the training process stable and manageable. Finally, we optimized the hardware deployment of DeepCAD-RT and achieved an overall improvement of a 27-fold reduction in memory consumption and a 20-fold acceleration in inference speed, which ultimately supported real-time image denoising once incorporated with the microscope acquisition system. We demonstrate the capability and generality of DeepCAD-RT on a series of photon-limited imaging experiments, including imaging calcium transients in various model organisms, such as mice, zebrafish and flies, observing the migration of neutrophils after acute brain injury and monitoring cortical neurotransmitter dynamics using a recently developed genetically encoded ATP sensor^34^.

## Results

### Comprehensive optimization of DeepCAD-RT for real-time processing

**Fig. 1:**
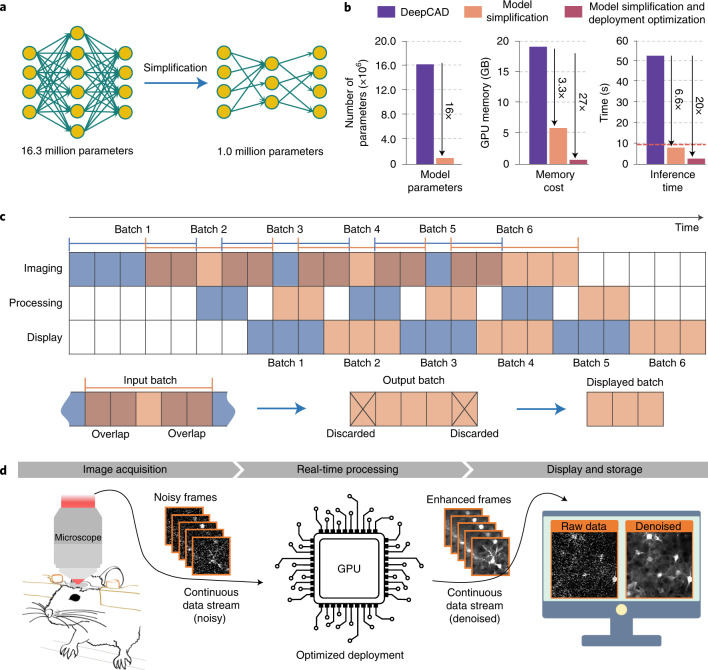
Optimization and real-time schedule of DeepCAD-RT. **a**, Model simplification by feature pruning. The total number of model parameters was reduced from ~16.3 million (16,315,585) to ~1.0 million (1,020,337) for higher processing speed and less memory consumption. **b**, Performance comparison between DeepCAD and DeepCAD-RT. Deployment optimization refers to hardware acceleration by further optimizing the deployment of deep neural networks on GPU cards. An example image sequence of 490 × 490 × 300 (*x*-*y*-*t*) pixels was partitioned into 75 patches (150 × 150 × 150 pixels with 40% overlap) to obtain these performance measurements on the same GPU (GeForce RTX 3090, Nvidia) with one batch size. In total, ~2.53 × 10^8^ pixels flowed through the network. All hyperparameters remained the same except the method. The red dashed line on the right indicates the imaging time (~9.6 s) of the example data; GB, gigabytes. **c**, Real-time schedule of DeepCAD-RT. The continuous data stream acquired from the microscope acquisition software was packaged into 3D (*x*-*y*-*t*) minibatches and fed into DeepCAD-RT. To maximize the processing speed, three parallel threads were programmed for image acquisition, data processing and display, respectively. For each batch, half of the overlap was discarded to avoid marginal artifacts. Overlapping frames between two consecutive batches are rendered with overlapping colors. **d**, Schematic of real-time denoising implemented with DeepCAD-RT on a two-photon microscope. Raw noisy data and the corresponding denoised data are displayed synchronously, which will be saved as separated files automatically at the end of the imaging session.

Limited by the computationally demanding nature of deep neural networks, the throughput of most deep learning-based methods for video processing is lower than the data acquisition rate^35^. To the best of our knowledge, no deep learning-based denoising methods for fluorescence imaging have been demonstrated to have real-time processing capability in practice. The original DeepCAD was proposed to denoise calcium imaging data in postprocessing. For the same amount of data, its processing time is about five times longer than the acquisition time. In this work, our rationale was to provide a compact and user-friendly tool that can be incorporated into the data acquisition pipeline to enhance the raw noisy data immediately after acquisition, which serves as the last step of data acquisition and the first step of data processing. Toward this goal, we started the first round of optimization by simplifying the network architecture (Fig. 1a). We compressed the network by pruning different proportions of network parameters and then investigated their performance using synthetic calcium imaging data simulated with neural anatomy and optical microscopy (NAOMi)^36^. Synthetic calcium imaging data have paired ground-truth images that are indispensable for rigorous comparison (Supplementary Fig. 1). Quantitative evaluation shows that although we removed as many as ~94% (from 16.3 million to 1.0 million) network parameters, the denoising performance did not deteriorate (Supplementary Fig. 2), while the memory cost and inference time were reduced by 3.3-fold and 6.6-fold, respectively, which pushed the processing throughput of the network to the same level as imaging (Fig. 1b). However, unlike denoising in postprocessing, real-time processing requires frequent data exchanges and necessitates extra computational resources for display and interaction. A practical processing throughput should be two to three times higher than imaging to reserve reasonable design margins. For further acceleration, we performed the second round of optimization in hardware deployment by implementing simplified models with TensorRT (Nvidia), a toolbox that provides optimized deployment of deep neural networks on specific graphics processing unit (GPU) cards. On our task, the deployment optimization reduced the memory cost and inference time by 8.2-fold and 3-fold, respectively. Combining model simplification and deployment optimization, the overall improvement is a 27-fold reduction in memory consumption and a 20-fold improvement in inference speed (Fig. 1b), making the implementation of real-time denoising possible.

**Extended Data Fig. 1:**
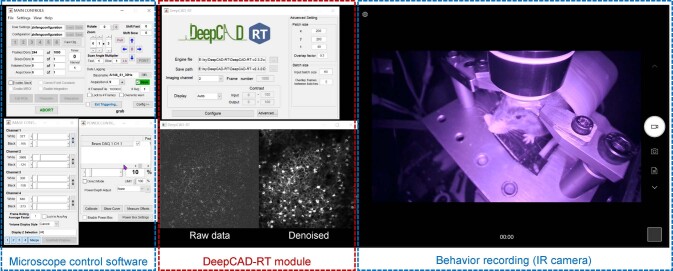
Real-time implementation of DeepCAD-RT. Real-time denoising was implemented by incorporating DeepCAD-RT into the image acquisition software. Images captured by the microscope were seamlessly fed into DeepCAD-RT, which denoised the input low-SNR images using pre-trained models and displayed denoised images after real-time processing.

To incorporate DeepCAD-RT into the data acquisition pipeline of the microscopy system, we designed three parallel threads for imaging, data processing and display (Fig. 1c). The continuous data stream captured by the microscope will be packaged into consecutive batches in the imaging thread and seamlessly fed into the processing thread. Once a new batch is received by the processing thread, the pretrained model already deployed on the GPU starts processing, and the denoised batch will be passed to the display thread. After removing overlapping frames, denoised batches will be assembled into a denoised stream and displayed on the monitor. The three threads keep temporally aligned throughout the whole imaging session. Both the raw noisy data and denoised data will be saved as separated files once the imaging session finishes. As a proof of concept, we demonstrate real-time denoising on a two-photon fluorescence microscope using DeepCAD-RT (Fig. 1d and Extended Data Fig. 1). The denoised data with drastically enhanced SNR can be presented simultaneously with the raw data (Supplementary Video 1), which facilitates the observation and evaluation of biological dynamics under photon-limited conditions.

**Extended Data Fig. 2:**
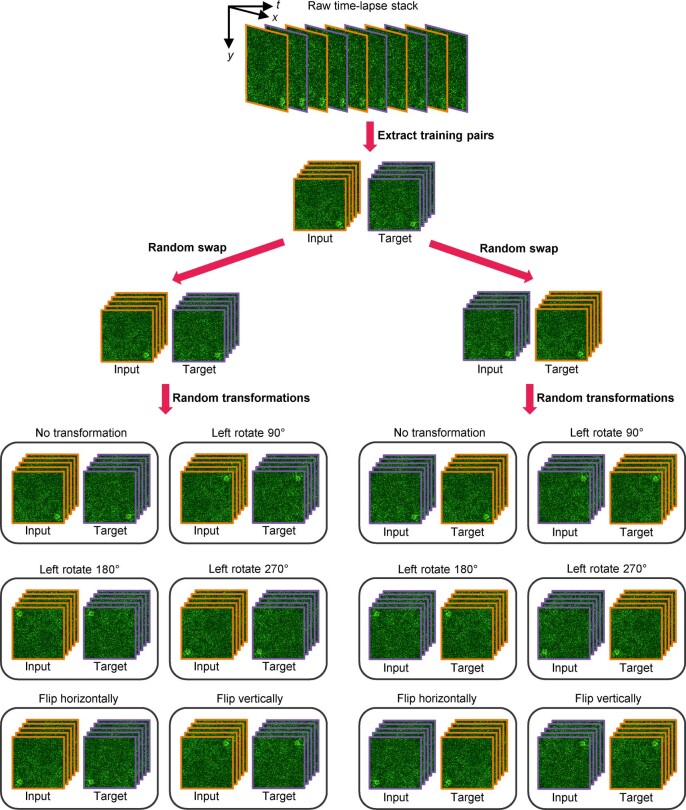
Data augmentation strategy. Adjacent frames in the original low-SNR stack (xy-t) were divided into two sub-stacks. One as the input volume and the other one as the target volume. Before being fed into the network for training, each training pair was augmented 12-fold through a random swap and six random geometric transformations.

**Extended Data Fig. 3:**
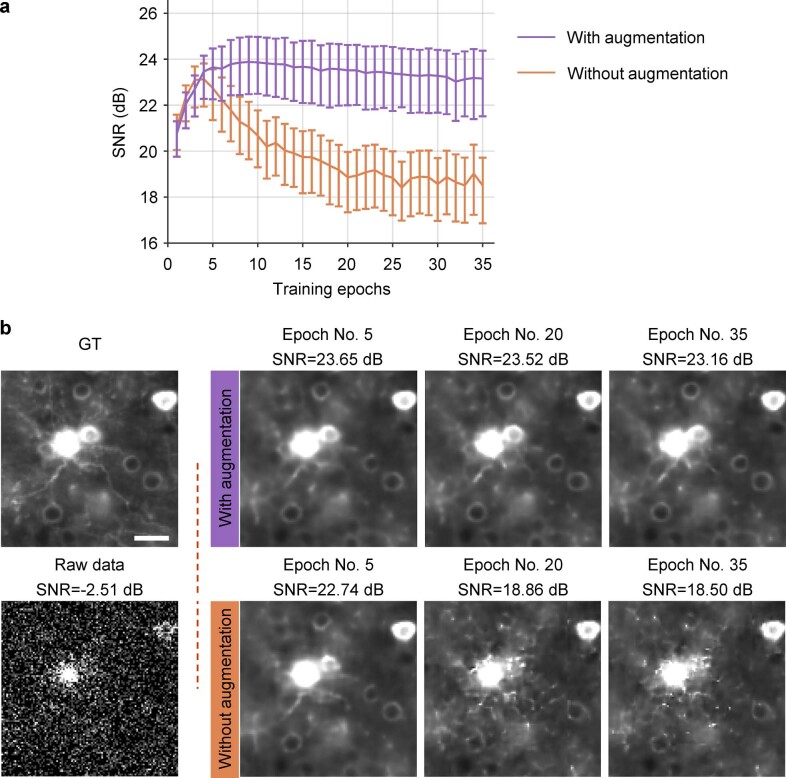
Training stability with and without data augmentation method. Simulated data (1000 frames, 30 Hz frame rate, SNR = −2.51 dB) were used in this experiment for quantitative evaluation. The network architecture has been simplified (~1.0 million trainable parameters). **a**, Denoising performance (SNR) with the increase of training epoch. Lines represent mean values and error bars represent the minimum and maximum values. **b**, Example ground truth (GT) images, raw data before denoising, and denoising results with and without data augmentation. Scale bar, 20 μm.

Besides real-time denoising, we also optimized the training procedure to make DeepCAD-RT easy to harness in various biological applications. We introduced 12-fold data augmentation (Extended Data Fig. 2) to reduce its data dependency. Currently, training the network with a low-SNR video stack containing as few as 1,000 frames is sufficient to ensure satisfactory performance (Supplementary Fig. 3). Moreover, we found that the combination of model simplification and data augmentation can effectively suppress overfitting (Extended Data Fig. 3), which was an inherent problem of self-supervised training and required human inspections for model selection previously^33^. We compared DeepCAD-RT with Noise2Void^37^ and Hierarchical DivNoising (HDN)^38^ and a supervised baseline (Methods), which shows that DeepCAD-RT performs very close to the supervised baseline and is much better than Noise2Void and HDN (Supplementary Figs. 4 and 5) because of its ability to integrate spatial and temporal correlations through the 3D architecture. We also compared DeepCAD-RT with DeepInterpolation, another recently developed denoising method leveraging interframe correlations^32^. The results indicate that, with the same amount of training data, DeepCAD-RT substantially outperformed DeepInterpolation, especially under photon-limited conditions (SNR < 5 dB). However, DeepCAD-RT can achieve comparable performance with tens of times less training data (trained from scratch with 6,000 frames) than DeepInterpolation (pretrained with 225,000 frames and then fine-tuned with 6,000 frames; Supplementary Figs. 4 and 5). The high data efficiency of DeepCAD-RT enables it to be extended to other applications beyond calcium imaging (Supplementary Fig. 6). In most cases, the data at hand can be directly used for training without requiring additional large-scale training datasets. Another advantage of DeepCAD-RT is that its processing speed can be at least an order of magnitude higher than DeepInterpolation even with the same network complexity and computational device because DeepCAD-RT outputs the entire three-dimensional (3D) stack from the 3D input, while DeepInterpolation just outputs a single frame from the 3D input.

### Denoising calcium imaging on multiple model organisms

**Extended Data Fig. 4:**
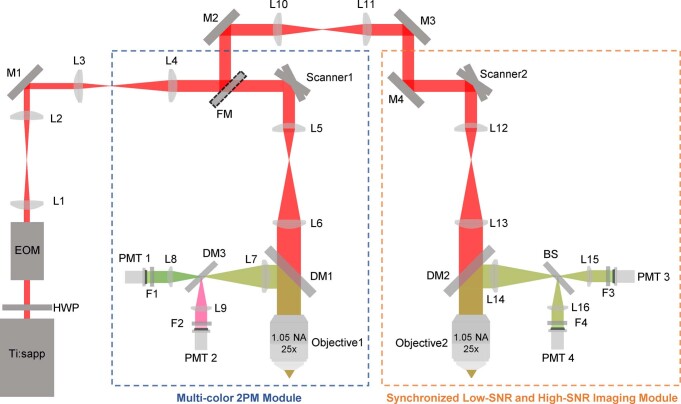
Imaging system. Our imaging system was composed of a multi-color two-photon module (blue box) and a custom-designed two-photon module to capture synchronized low-SNR and high-SNR images (yellow box). Ti:sapp: titanium-sapphire laser with tunable wavelength; HWP: half-wave plate; EOM: electro-optic modulator; M1-M4: mirrors; L1-L16: lens; Scanner1, Scanner2: galvo-resonant scanners; DM1, DM2: long-pass dichroic mirrors to separate fluorescence signals (green path) from the excitation laser (red path); DM3: short-pass dichroic mirror to separate green fluorescence and red fluorescence. FM: flip mount to alternate between the two modules; F1-F4: emission filters; BS: 1:9 (reflectance: transmission) non-polarizing plate beam splitter; PMT1-PMT4: photomultiplier tubes.

**Extended Data Fig. 5:**
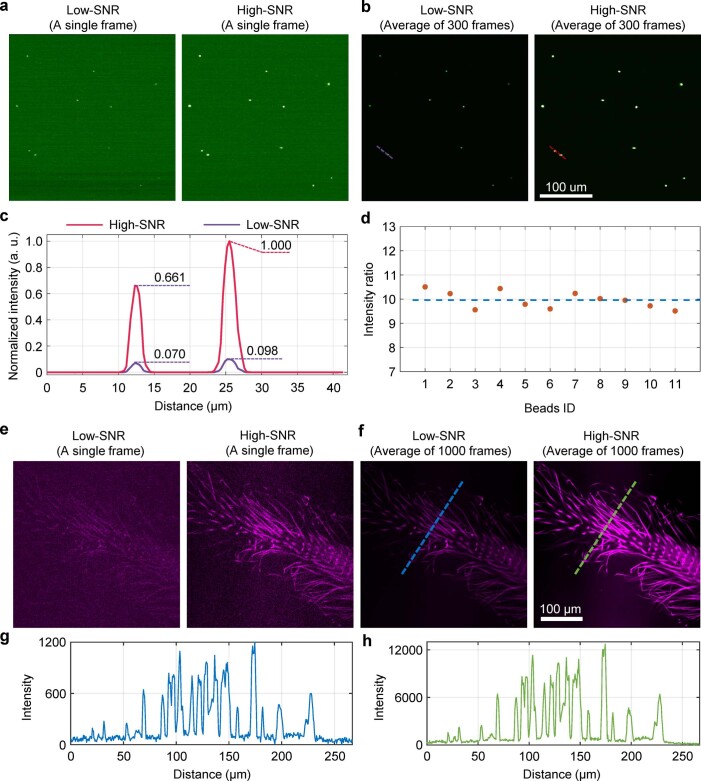
System calibration. **a**, Example frames captured by the low-SNR detection path (left) and the high-SNR detection path (right). **b**, Average projection of 300 continuously acquired frames. Noise was largely suppressed and underlying fluorescence signals were revealed. **c**, Intensity profiles (normalized to the maximum of high-SNR recording) along the dashed lines in **b**. **d**, The intensity (photon) ratios (high-SNR relative to low-SNR) of all 11 fluorescent beads in the FOV. Each point represents one bead and the average intensity ratio is ~10.0 (blue dashed line). **e**, Example images of an insect slice captured by the low-SNR detection path (left) and the high-SNR detection path (right). **f**, Average projection of 1000 consecutive frames. **g**, **h**, Intensity profiles along the blue and green dashed lines in **f**.

Although synthetic data can provide ground-truth images that are not experimentally available, the performance of denoising methods should be quantitatively evaluated with experimentally obtained data for best reliability. Motivated by this principle, we captured synchronized low-SNR and high-SNR image pairs with our custom-designed two-photon microscope (Extended Data Fig. 4) for each type of experiment. The low-SNR data were used as the input, while the synchronized high-SNR data with tenfold fluorescence photons were used for result validation (Extended Data Fig. 5). A standard two-photon microscope was also integrated into our system for cross-system validation and multicolor imaging.

**Fig. 2:**
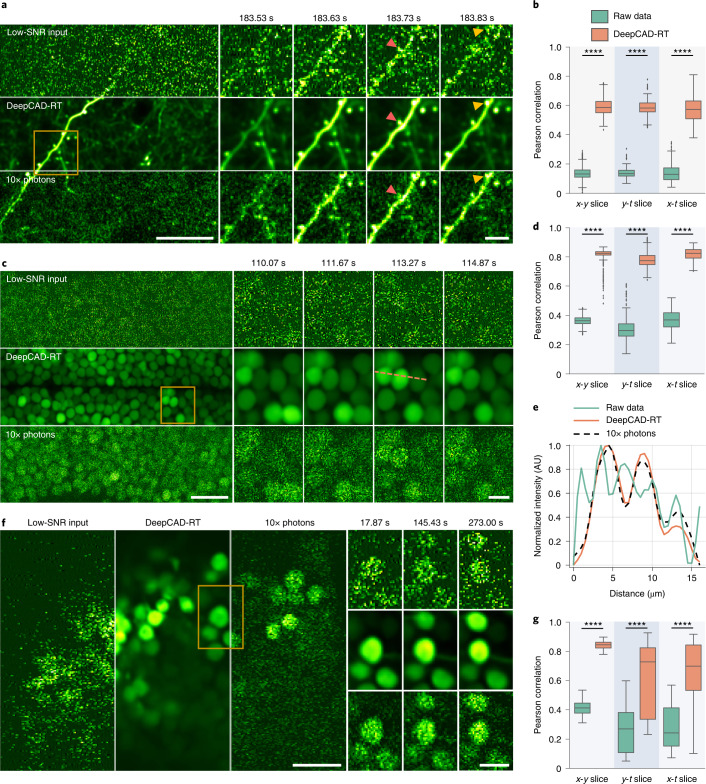
Universal denoising of calcium imaging in mice, zebrafish and *Drosophila*. **a**, Imaging calcium transients in dendritic spines of a mouse expressing genetically encoded GCaMP6f calcium indicator. One example frame is shown for the low-SNR raw recording (top), DeepCAD-RT denoised recording (middle) and synchronized high-SNR recording with tenfold fluorescence photons (bottom). Magnified views of the yellow boxed region show calcium dynamics of two spatially adjacent dendritic branches. Each frame was integrated for 33 ms to ensure high temporal resolution. Red arrowheads point to a mushroom spine, and yellow arrowheads point to a stubby spine; scale bars, 20 μm for the whole FOV and 5 μm for magnified views. **b**, Box plots showing image correlations along three dimensions (*x*-*y*-*t*) before and after denoising. The high-SNR data with tenfold fluorescence photons were used as the reference for correlation computing; *x-y* slice, *N* = 6,000; *y-t* slice, *N* = 246; *x-t* slice, *N* = 489. **c**, Time-lapse imaging of calcium dynamics of optic tectum neurons in the zebrafish brain (HuC:GCaMP6s). Top, the original low-SNR data. Middle, DeepCAD-RT enhanced data. Bottom, high-SNR recording with tenfold photons. Magnified views show the neural activity of the yellow boxed region in a short period. Each frame was integrated for 66 ms; scale bars, 20 μm for the entire FOV and 5 μm for magnified views. **d**, Pearson correlations of image slices along three dimensions before and after denoising; *x-y* slice, *N* = 6,000; *y-t* slice, *N* = 246; *x-t* slice, *N* = 246. **e**, Intensity profiles of the yellow dashed line in **c**. Pixel intensities were extracted from twofold downsampled images, and all traces were smoothed by moving average with a 3-pixel kernel to suppress the noise; AU, arbitrary units. **f**, Denoising performance of DeepCAD-RT on calcium imaging of *Drosophila* mushroom bodies (GCaMP7f). The same frame is shown for the original low-SNR data (left), DeepCAD-RT denoised image (middle) and high-SNR image with tenfold fluorescence photons (right). Magnified views show snapshots of the yellow boxed region at three moments. Each frame was integrated for 33 ms; scale bars, 10 μm for the whole FOV and 5 μm for magnified views. **g**, Box plots showing the improvement of image correlation after denoising; *x-y* slice, *N* = 12,000; *y-t* slice, *N* = 241; *x-t* slice, N = 335. Asterisks denote significance levels tested with one-sided paired *t*-test; ****P < 0.0001 for all comparisons. Source data

To demonstrate the capability and generality of our method, we first investigated whether it could be applied to various calcium imaging experiments. We began by imaging calcium transients of postsynaptic dendritic spines in cortical layer 1 (L1) of a mouse expressing genetically encoded GCaMP6f^39^. Technically, calcium imaging of dendritic spines over a large field-of-view (FOV) is particularly challenging because of their small sizes^40^. Each spine is usually characterized by as few as several pixels, and noise severely contaminates its spatiotemporal features. After we enhanced the original low-SNR data with our method, the image SNR was substantially improved, and postsynaptic structures can be clearly resolved even in a single frame (Fig. 2a and Supplementary Video 2). Without noise contamination, the morphological heterogeneity between mushroom spines and stubby spines became discernable. Because different spine classes have different functions during development and learning^41^, revealing spine morphology is helpful for the study of dendritic computing. For quantitative evaluation, we extracted image slices along three dimensions (*x*-*y*-*t*) and calculated image correlations with corresponding high-SNR images. Statistical analysis shows that image correlations can be significantly improved for all three dimensions after denoising (Fig. 2b), manifesting the spatial and temporal denoising capability of our method.

**Extended Data Fig. 6:**
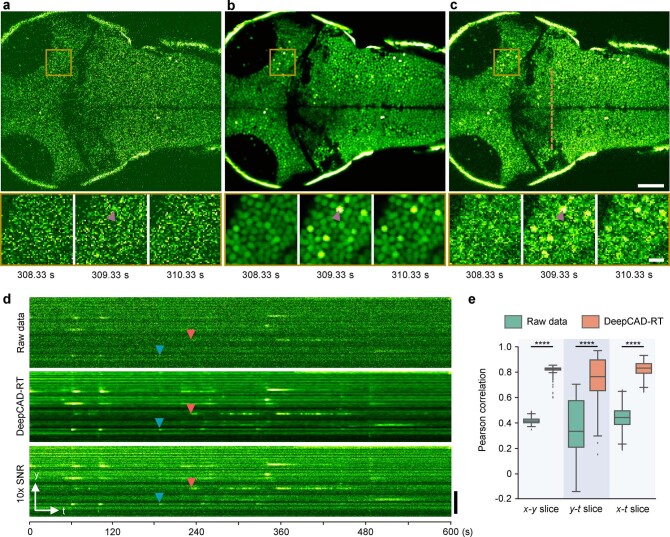
Denoising calcium imaging across multiple brain regions in larval zebrafish. **a**, Original low-SNR recording. **b**, DeepCAD-RT enhanced data. **c**, Synchronous high-SNR recording with 10-fold fluorescence photons. Magnified views of the yellow boxed region showing calcium dynamics in a 2-second period. Arrowheads point to the same neuron. Scale bar, 50 μm for the large FOV and 10 μm for magnified views. **d**, *y-t* slices along the dashed line in **c**. Two calcium events are indicated with arrowheads of different colors. Scale bar, 50 μm. **e**, Pearson correlation of image slices along all three dimensions before and after denoising. *x-y* slice, N = 9000; *y-t* slice, N = 400, *x-t* slice, N = 485. P values were calculated by one-sided paired t-test. ****P < 0.0001 for all comparisons. Source data

Animal models currently used in systems and evolutionary neuroscience are diverse and extend from jellyfish^42^ to monkeys^43^. To test our method on versatile animal models with different neuron morphologies and brain structures, we imaged in vivo calcium dynamics in the brains of zebrafish larvae and *Drosophila* and denoised the original shot-noise-limited signals with our method. For zebrafish imaging, we used larval zebrafish expressing nuclear-localized GCaMP6s calcium indicator throughout the whole brain. Because of the shot noise, raw images deteriorated severely, and neurons can be barely recognized. However, after denoising, the image SNR was massively improved, and fluorescence signals became clear (Fig. 2c and Supplementary Video 3). Image correlations along all three dimensions were significantly improved (Fig. 2d). In each frame, the distribution of optic tectum neurons can be clearly recognized with the enhancement of our method (Fig. 2e). Additionally, we also imaged calcium events of large neuronal populations spanning multiple brain regions and found that the removal of noise was rather helpful for separating densely labeled cells. (Extended Data Fig. 6 and Supplementary Video 4). Similarly, we performed time-lapse calcium imaging of mushroom body neurons in the brains of adult *Drosophila*. The results showed that the enhanced imaging SNR and image correlations could facilitate the observation of calcium dynamics (Fig. 2f,g and Supplementary Video 5), which verified the effectiveness of our method on various calcium imaging applications involving different animal models and neuronal structures. Because smaller animals such as zebrafish and *Drosophila* are less resistant to high excitation power than mice, it is difficult to keep the sample healthy and obtain high-SNR imaging data simultaneously. With its good performance and versatility, DeepCAD-RT can be a promising tool for calcium imaging to minimize the excitation power and photon-induced disturbance by removing shot noise computationally.

### Observing neutrophil migration in vivo with low excitation power

**Fig. 3:**
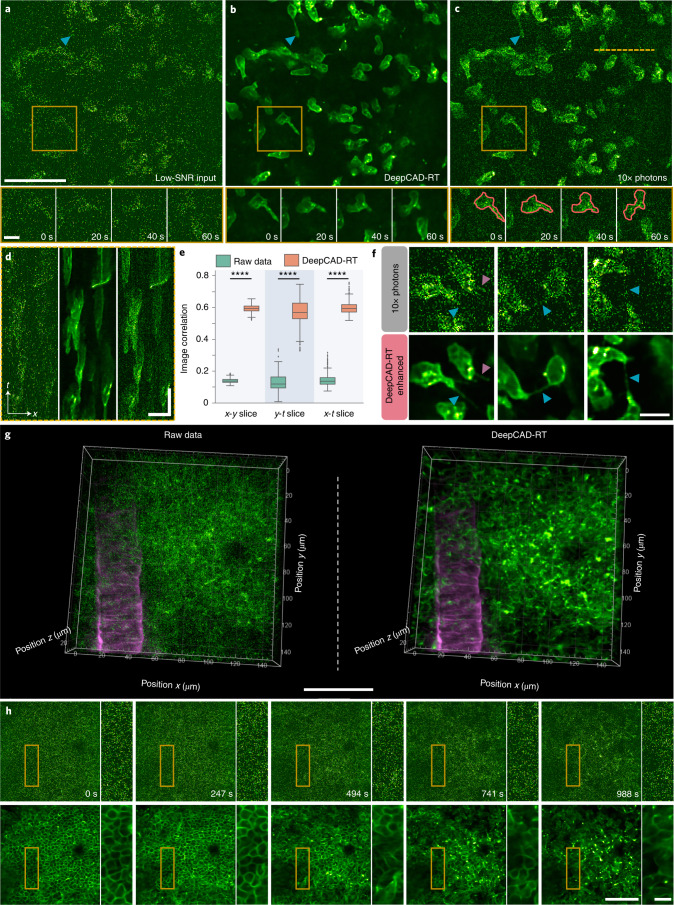
Observing 3D migration of neutrophils in the mouse brain in vivo. **a**, Low-SNR images of neutrophil migration without denoising. **b**, Images denoised with DeepCAD-RT. **c**, Synchronized high-SNR images with tenfold fluorescence photons. Blue arrowheads point to the elongated tail of a migrating neutrophil. Magnified views of the yellow boxed region showing the morphological evolution of neutrophils in a 60-s time window. Red closed lines annotate the border of a neutrophil during migration. Neutrophils were labeled with a fluorescent-conjugated Ly-6G antibody. Each frame was integrated for 100 ms, and the entire time-lapse imaging session lasted 644 s; scale bars, 50 μm for the whole FOV and 10 μm for magnified views. **d**, *x-t* slices along the yellow dashed line in **c** of low-SNR raw data (left), DeepCAD-RT denoised data (middle) and corresponding high-SNR data with tenfold fluorescence photons (right); scale bars, 20 μm for *x* and 50 s for *t*. **e**, Box plots showing Pearson correlations of image slices along three dimensions (*x*-*y*-*t*) before and after denoising; *x*-*y* slice, *N* = 6,440; *y*-*t* slice, *N* = 512; *x*-*t* slice, *N* = 512. *P* values were calculated by one-sided paired *t*-test; ****P < 0.0001 for all comparisons. **f**, Denoising high-SNR data with DeepCAD-RT reveals subcellular dynamics of neutrophils. Retraction fibers are indicated with arrowheads; scale bar, 10 μm. **g**, Three-dimensional imaging of neutrophil migration in a 150 × 150 × 30 μm^3^ volume (15 planes) after acute brain injury. The raw noisy volume (left) and corresponding denoised volume (right) are visualized from the same perspective. Acute brain injury was induced by craniotomy. Neutrophils were labeled with a fluorescent-conjugated Ly-6G antibody (green channel). Blood vessels were stained with a WGA (magenta channel) dye. Because blood vessels are stationary, noise in the magenta channel was removed by averaging multiple frames; scale bar, 50 μm. **h**, Images of a single plane before (top) and after (bottom) denoising. DeepCAD reveals diffusion of the neutrophil population. Magnified views of yellow boxed regions are shown next to each image; scale bars, 50 μm for the entire FOV and 10 μm for magnified views. Source data

Our previous work only focused on calcium imaging, in which neurons are spatially invariant and their intensity changes over time. Next, we applied our method to the observation of cell migration, a complementary task with almost temporally invariant intensity and continuously changing cell positions. Neutrophils are the most abundant white blood cells in immune defense^44^. To fully understand the function of neutrophils, intravital imaging with minimal illumination is essential because phototoxicity and photodamage would alter cellular and subcellular processes, which potentially disturb normal immune responses^16,45^. We first evaluated the performance of our method on cell migration observations qualitatively and quantitatively with synchronized low-SNR and high-SNR (tenfold photons) image pairs captured by our customized system. The results showed that DeepCAD-RT can restore neutrophils of different shapes from noise and the evolution of morphological features over time (Fig. 3a–c and Supplementary Video 6). Because the SNR of denoised data is better than high-SNR data with tenfold photons, the illumination power can be equivalently reduced more than tenfold for linear microscopy and more than threefold for two-photon microscopy. For better comparison, we show the kymographs (*x*-*t* projections) of marked regions. The migration of neutrophils could be visualized directly in denoised data rather than submersed in noise in low-SNR raw data (Fig. 3d). Quantitative evaluation also indicated that denoised data are more correlated to high-SNR data (Fig. 3e). Additionally, the substantial improvement of image SNR after denoising prompted us to investigate whether our method could reveal more cellular traits if it took high-SNR data as the input. After training and inference with the high-SNR data, we found that higher input SNR could produce much better denoising results. The dynamics of retraction fibers during neutrophil migration could be visualized after the enhancement of our method (Fig. 3f and Supplementary Video 7).

**Extended Data Fig. 7:**
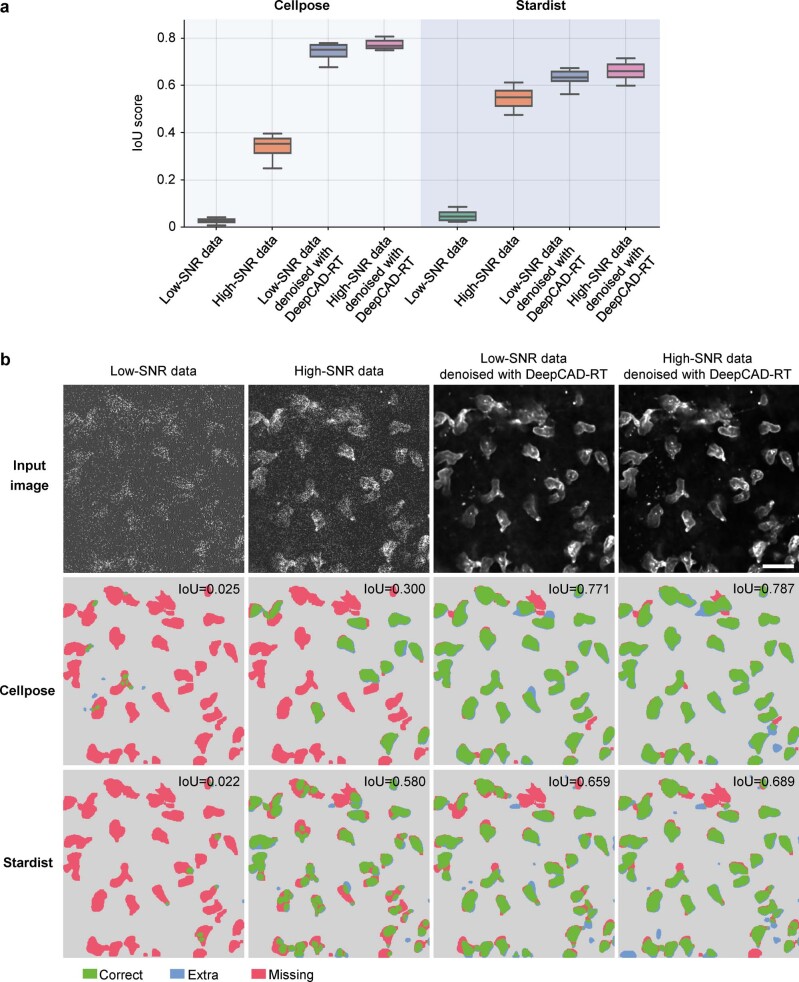
The performance of neutrophil segmentation before and after denoising. **a**, Segmentation performance of Cellpose^46^ and Stardist^47^ on raw low-SNR data, synchronous high-SNR data (10-fold fluorescence photons), DeepCAD denoised raw data, and DeepCAD denoised high-SNR data (N = 10). The Intersection-over-Union (IoU) score was used to quantify the segmentation performance. Manually annotated masks were used as the ground truth. **b**, Representative input images and segmented masks. Correctly segmented regions (true positive) are colored green. Missing (false negative) and extra regions (false positive) are colored red and blue, respectively. Scale bar, 20 μm. Source data

For fluorescence microscopy, denoising is the first step of subsequent data processing and downstream biological analysis. A good denoising method can facilitate cell segmentation, localization and classification, which are fundamental steps for the study of cell migration. To figure out the improvement our method brings to segmentation, we segmented neutrophils from the original noisy images (both low-SNR and high-SNR) and corresponding denoised images using Cellpose^46^ and Stardist^47^, two recently published methods for cellular segmentation with state-of-the-art performance^48^. We enlisted five expert human annotators to manually label cell borders and obtain ground-truth masks through majority voting (Methods). Using intersection-over-union (IoU) score as the metric, the segmentation performance of the two methods could be improved by ~30-fold for low-SNR images (Extended Data Fig. 7). For high-SNR images with tenfold fluorescence photons, we also observed a substantial improvement for both methods because shot noise was removed, and cell structures could be well recognized after denoising.

The migration of neutrophils is coordinated in 3D. Deciphering its spatiotemporal pattern necessitates volumetric imaging. Using our multicolor two-photon microscope, we imaged a 150 × 150 × 30 μm^3^ volume in the mouse brain after acute brain injury induced by craniotomy. The volume rate of the entire imaging session was 2 Hz. Fluorescence signals from neutrophils and blood vessels were recorded simultaneously and merged into multicolor images post hoc. To minimize the interference caused by the excitation laser and record the native pattern of neutrophil migration, the excitation power we used was below 30 mW. Because the fluorescence labeling of neutrophils was only localized to their membranes, the concentration of the fluorophore was low. The SNR of the raw data was very low, and cell structures and dynamics could not be visualized because of the contamination of shot noise (Fig. 3g). After we denoised these low-SNR raw data with our method, shot noise was effectively suppressed, and the 3D dynamics of neutrophil migration became explicit (Supplementary Video 8), which unveiled the phenomenon that a cluster of neutrophils congregating in the early stage of inflammation diffused over time (Fig. 3h).

### DeepCAD-RT facilitates the recording of neurotransmitter dynamics

**Fig. 4:**
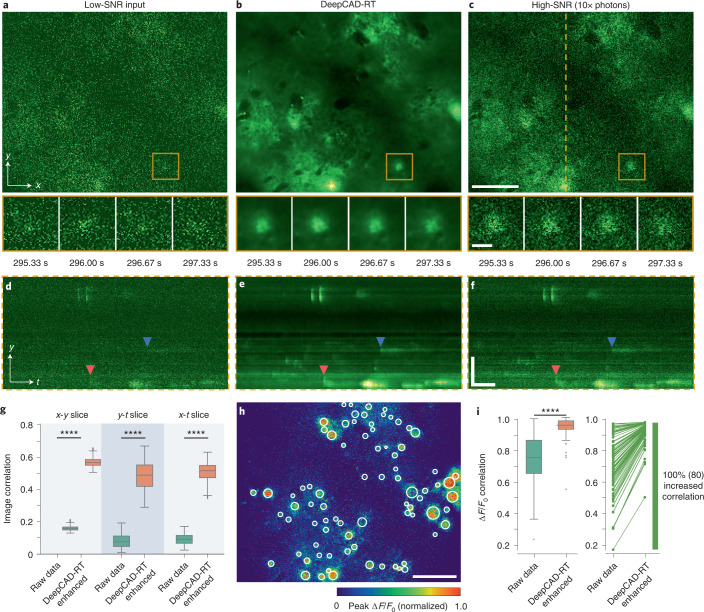
Denoising performance of DeepCAD-RT on neurotransmitter imaging in live mice. **a**, Low-SNR recording of extracellular ATP release in the mouse brain. **b**, DeepCAD-RT enhanced data with low-SNR recording as the input. **c**, Synchronized high-SNR data with tenfold photons. Magnified views showing ATP dynamics in the yellow boxed region in a 2-s period. Each frame was integrated for 67 ms; scale bars, 50 μm for the large FOV and 10 μm for magnified views. **d**–**f**, *y*-*t* slices along the dashed line in **c**. Two ATP release events are indicated with arrowheads of different colors; scale bars, 50 μm for *y* and 50 s for *t*. **g**, Pearson correlation coefficients of *x*-*y*, *y*-*t* and *x*-*t* slices before and after denoising; *x*-*y* slice, *N* = 7,000; *y*-*t* slice, *N* = 476; *x*-*t* slice, *N* = 476. **h**, Peak Δ*F*/*F*_0_ (*F* is the fluorescence trace and *F*_0_ is the baseline fluorescence approximated by the average of the entire trace) of high-SNR data with tenfold fluorescence photons during the whole imaging session (~480 s). Manually annotated release sites are marked with white circles (*N* = 80); scale bar, 50 μm. **i**, Left, box plots showing Pearson correlations of fluorescence traces extracted from release sites in **h** before and after denoising (*N* = 80). Traces extracted from high-SNR data with tenfold photons were used as the ground truth for correlation calculation. Right, increases of trace correlation. Each line represents 1 of 80 traces, and increased correlations are colored green. *P* values calculated by one-sided paired *t*-test are specified with asterisks; ****P < 0.0001 for all comparisons. Source data

With the recent proliferation of different fluorescent indicators, combining fluorescence microscopy and genetically encoded fluorescent indicators has become a widespread methodology for interrogating the structural, functional and metabolic mechanisms of living organisms^49^. For the nervous system alone, available activity indicators have gone beyond calcium and already extended to other intracellular and extracellular neurotransmitters, including dopamine^50,51^, GABA (γ-aminobutyric acid)^52^, glutamate^53,54^, acetylcholine^26,55^ and so on. Similar to calcium imaging, shot noise is also a restriction for the imaging of other activity sensors, which reduces the image SNR and limits in vivo characterization and applications. To investigate whether our method can be extended to neurotransmitter sensors, we took an ATP sensor as an example and recorded cortical ATP release using mice expressing GRAB_ATP1.0_ (ref. ^34^), a recently developed genetically encoded sensor for measuring extracellular ATP (Methods). In the low-SNR raw data, shot noise swamped ATP signals (Fig. 4a). After denoising with our method, these release events were clearly visualized (Fig. 4b,c and Supplementary Video 9). Kymographs (*y*-*t* projections) showed that some subtle ATP release events that could be omitted in the raw data become visible (Fig. 4d–f). Quantitatively, we used corresponding high-SNR images as the ground truth to calculate image correlations along all three dimensions and found that image correlations could be significantly improved after denoising (Fig. 4g). To compare ATP traces before and after denoising, we manually annotated 80 firing sites from the heat map of peak Δ*F*/*F*_0_ (Fig. 4h) and extracted fluorescence traces representing ATP activity over time. We calculated Pearson correlations between all traces and the ground truth (traces extracted from the high-SNR data). Statistical results showed that the signals of ATP release can be effectively enhanced, and the correlations of all fluorescence traces are improved, benefiting from the removal of noise (Fig. 4i).

**Fig. 5:**
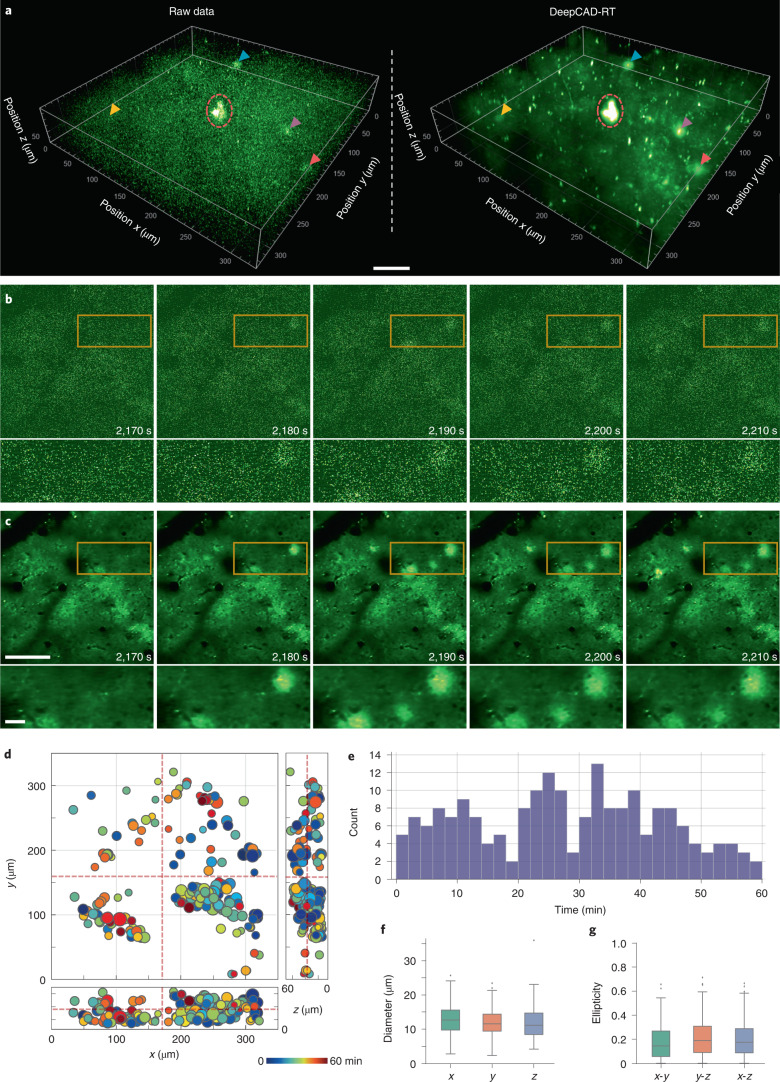
DeepCAD-RT reveals the spatiotemporal patterns of extracellular ATP in vivo after laser-induced brain injury. **a**, Three-dimensional visualization of ATP release events in a 350 × 350 × 60 μm^3^ volume (30 planes, 1-Hz volume rate) after laser-induced brain injury. Left, low-SNR raw volume without denoising. Right, the same volume enhanced with DeepCAD-RT. A representative moment is demonstrated here, and similar performance was achieved throughout the whole imaging session (1 h, 3,600 volumes). Four ATP release events are indicated with arrowheads of different colors. The laser-ablated point (red dashed circle) was located at the center of the volume; scale bar, 50 μm. **b**, Example raw frames of a single plane at four different time points. **c**, DeepCAD-RT enhanced frames corresponding to those in **b**. Magnified views of yellow boxed regions are shown under each image; scale bars, 100 μm for the whole FOV and 20 μm for magnified views. **d**, The spatiotemporal distribution of ATP release during the 1-h-long recording. The release time is color coded, and the diameter of each release event scales to the size of each circle. The intersections of red dashed lines indicate the 3D location of the laser-induced injury. **e**, Counting ATP release events along the time axis. The binning width is 2 min. **f**, Box plots showing diameters of all release events (*N* = 196) in three orthogonal dimensions; *x*, 13.131 ± 0.3090; *y*, 12.125 ± 0.2911; *z*, 11.907 ± 0.3287 (mean ± s.e.m.). **g**, Statistics on the ellipticity of all release events (*N* = 196) in three orthogonal coordinate planes; *x*-*y*, 0.182 ± 0.0109; *y*-*z*, 0.213 ± 0.0114; *x*-*z*, 0.205 ± 0.0109 (mean ± s.e.m.). Source data

Previous studies on in vivo imaging of ATP release were restricted in two-dimensional (2D) planes^34,56^. To fully unveil the spatiotemporal distribution and evolution pattern of ATP release in 3D tissues, we performed volumetric imaging of a 350 × 350 × 60 μm^3^ tissue volume in the mouse brain after laser-ablated injury. The injury site was located at the center of the volume. Because inflammation and injury can trigger the release of endogenous ATP, phototoxicity and photodamage caused by the excitation laser should be minimized to avoid undesired disturbance. Thus, we kept the excitation power below 40 mW and imaged the 3D volume continuously for 1 h. In the shot-noise-limited raw data, noise was dominant, and only a few intense events were seen (Fig. 5a). To suppress the shot noise and visualize as many release events as possible, we trained a denoising model with our method and enhanced the original low-SNR data. Denoised data had very high SNR, and those release events concealed by noise turned out to be discernable (Fig. 5a and Supplementary Video 10). For better comparison, we present several snapshots of a single plane at different moments (Fig. 5b,c), which indicates the superior denoising performance of our method. We manually annotated the position and time of all ATP release events throughout the entire session (Fig. 5d) and found that the release frequency is approximately random during the 1-h imaging (Fig. 5e). Owing to the noise removal capability, the spatial profile of ATP release was clarified, and performing statistics on their geometric features (diameter and ellipticity) became feasible (Fig. 5f,g). The successful extension of DeepCAD-RT to the imaging of ATP release indicates its good potential on other neurotransmitter sensors.

## Discussion

Noise is an ineluctable obstacle in scientific observation. For fluorescence microscopy, the inherent shot-noise limit determines the upper bound of imaging SNR and restricts imaging resolution, speed and sensitivity. In this work, we present a versatile method to denoise fluorescence images with rapid processing speed that can be incorporated with a microscope acquisition system to achieve real-time denoising. Our method is based on deep self-supervised learning, and the original low-SNR data can be directly used for training convolutional networks, making it particularly advantageous in functional imaging where the sample is undergoing fast dynamics, and capturing ground-truth data is hard or impossible. We have demonstrated extensive experiments, including calcium imaging in mice, zebrafish and flies, cell migration observations and the imaging of a new genetically encoded ATP sensor, covering both 2D single-plane imaging and 3D volumetric imaging. Qualitative and quantitative evaluations show that our method can substantially enhance fluorescence time-lapse imaging data and permit high-sensitivity imaging of biological dynamics beyond the shot-noise limit.

Removing shot noise from fluorescence images promises to catalyze advancements in several imaging technologies. For example, in two-photon microscopy, multiplexed excitation by multiple laser foci can increase imaging speed, but the imaging SNR will decrease quadratically because of dispersed excitation power^57–59^. Our denoising method provides a potential solution to compensate for the SNR loss. Three-photon microscopy can effectively suppress background fluorescence and improve imaging depth through three-order non-linear excitation and longer wavelength^60,61^, but its practical use in deep tissue is still limited by low imaging SNR. Combining our method with three-photon microscopy could expedite its application in the deep mammalian brain. Light-field microscopy is an emerging technique for fast volumetric imaging of biological dynamics, but it relies on computational reconstruction that is sensitive to noise^62–64^. Disentangling underlying signals from noisy images before light-field reconstruction could eliminate artifacts and ensure high-fidelity results. Moreover, a recently published work reported that standard Richardson–Lucy deconvolution can recover high-frequency information beyond the spatial frequency limit of the microscope if there is no noise contamination^65^, which inspires us that our method would be helpful for deconvolution algorithms by denoising input images in advance. Single-molecule localization microscopy is also susceptible to noise because the localization precision is fundamentally limited by SNR^3,66^. The noise-sensitive nature holds for other super-resolution microscopy techniques, such as stimulated emission depletion microscopy and structured illumination microscopy^67,68^. We reasonably envisage that our method and its future variants would benefit the development of super-resolution microscopy.

As the backbone of our method lies in deep learning, its content-dependent trait requires users to train a specialized model for each task or each type of sample to ensure optimal results. Developing pretrained models on large-scale datasets and transferring them to new tasks by fine-tuning could be an optional solution to this problem. Another limitation is that adjacent frames used for training should have approximately identical underlying signals, which is the basic assumption of our self-supervised training strategy. Thus, the imaging system should have adequate temporal resolution relative to the biological dynamics to be imaged. Finally, the denoising performance of our method improves as the SNR of the input data increases. Comprehensive noise suppression by collaborating physics-based approaches^20,29^ and computational denoising could be a way to achieve higher imaging sensitivity beyond the shot-noise limit.

## Methods

### Imaging system

The optical setup integrated two two-photon microscopes for different purposes. One was a standard two-photon microscope with multicolor detection capabilities for multilabeling imaging and cross-system validation. The other was a custom-designed two-photon microscope to capture synchronized low-SNR and high-SNR (tenfold fluorescence photons) images for result validation (Extended Data Fig. 4). The two systems shared a titanium-sapphire femtosecond laser source with tunable wavelength (Mai Tai HP, Spectra-Physics). The excitation laser for all experiments was a linearly polarized Gaussian beam with a 920-nm central wavelength and an 80-MHz repetition rate. Before being projected into both systems, the laser beam was first adjusted in polarization by a half-wave plate (AQWP10M-980, Thorlabs) and modulated in intensity by an electro-optic modulator (350-80LA-02, Conoptics). A 1:1 4f system composed of two achromatic convex lenses (AC508-100-B, Thorlabs) was then configured to collimate the laser beam. Another 1:4 4f system (AC508-100-B and AC508-400-B, Thorlabs) was followed to expand the diameter of the beam. A mirror mounted on a two-position, motorized flip mount (MFF101, Thorlabs) was used to alternate between the two systems (OFF for the multicolor module and ON for the custom module).

The two systems used the same optical configuration for two-photon excitation. Specifically, the collimated, scaled laser beam was successively guided onto the fast axis (the resonant mirror) and the slow axis (the galvanometric mirror) of the galvo-resonant scanner (8315K/CRS8K, Cambridge Technology). The scanner provided fast 2D raster scanning under the control of two voltage signals. The orientation of the incident beam should be fine-adjusted to ensure the horizontality of the outgoing beam. Then, the output beam was recollimated, rescaled and corrected by a scan lens (SL50-2P2, Thorlabs) and a tube lens (TTL200MP, Thorlabs) to fit the back pupil of the objective and produce a flat image plane. We used a high numerical aperture (NA) water-immersion objective (×25/1.05-NA, XLPLN25XWMP2, Olympus) to expand the detection angle and increase the number of photons that can be detected. Approximately, the effective excitation NA was 0.7 in our experiments. To perform 3D volumetric imaging, we mounted the objective on a piezoelectric actuator (P-725, Physik Instrumente) to achieve high-precision axial scanning. For the detection path of the standard multicolor system, fluorescence photons emitted from the sample were captured by the objective and separated from the excitation light by a long-pass dichroic mirror (DMLP650L, Thorlabs). Another short-pass dichroic mirror (DMSP550, Thorlabs) was mounted in the detection path to separate green fluorescence and red fluorescence. The green fluorescence was purified by a pair of emission filters (MF525-39, Thorlabs; ET510/80M, Chroma) and detected by a GaAsP photomultiplier tube (PMT; H10770PA-40, Hamamatsu). The red fluorescence was filtered by an emission filter (ET585/65M, Chroma) and detected by the same type of PMT. For the detection path of the customized system for simultaneous low-SNR and high-SNR imaging, the previously mentioned short-pass dichroic mirror was replaced with a 1:9 (reflectance:transmission) non-polarizing plate beam splitter (BSN10, Thorlabs). Low-SNR images were formed by the ~10% reflected photons, and high-SNR images were formed by the ~90% transmitted photons. In this system, only green fluorescence was detected, and the same filters and PMT were used for both the low-SNR and high-SNR detection paths. The sensor plane of each PMT was conjugated to the back pupil plane of the objective using a 4:1 4f system (TTL200-A and AC254-050-A, Thorlabs) to maximize the detection efficiency. In general, the maximum FOV of the two two-photon microscopes was about 720 μm. The typical frame rate was 30 Hz for 512 × 512 pixels, and the volume rate decreased linearly with the number of planes to be scanned.

### System calibration

We imaged green-fluorescent beads to calibrate our imaging systems. For sample preparation, the original bead suspension was first diluted and embedded in 1.0% agarose and mounted on microscope slides to form a single bead layer composed of sparsely distributed beads. We calibrated both systems using 0.2-μm fluorescent beads (G200, Thermo Fisher) to obtain the lateral and axial resolution. Because the two systems had identical excitation optics, they had the same optical resolution. The lateral full width at half maximum (FWHM) is ~0.6 μm, and the axial FWHM is ~3.5 μm (Supplementary Fig. 7). To calibrate the intensity ratio between the high-SNR detection path and the low-SNR detection path, we imaged 1-μm fluorescent beads (G0100, Thermo Fisher) and found that the intensity ratio is about 1:10 (Extended Data Fig. 5a–d), which indicated that the number of fluorescence photons of the high-SNR detection path was about ten times higher than that of the low-SNR detection path. High-SNR data synchronized with low-SNR data could serve as a reference to unveil underlying signals. We also imaged insect slices for validation, and the results confirmed our calibration (Extended Data Fig. 5e–h).

### Model simplification

Theoretically, large models with more trainable parameters can implement extremely intricate functions on the input data. However, the very big model (16,315,585 (abbreviated 16.3 million) parameters in total) we previously used caused a series of problems, such as long training and inference time, large memory consumption and serious overfitting. We sought to solve these problems by simplifying the network architecture. Because network depth is of crucial importance for the performance^69^, instead of changing the depth of the network, we turned to reduce the number of feature maps in each convolutional layer. By continuously halving network parameters, we constructed seven models with exponentially decreased trainable parameters (16.3 million, 9.2 million, 4.1 million, 2.3 million, 1.0 million, 0.57 million and 0.26 million, respectively). To evaluate these models, we used synthetic calcium imaging data of −2.5 dB SNR and trained them with the same amount of data (6,000 frames). The best training epoch of each model was determined by monitoring its performance on a validation set. Although the number of trainable parameters was reduced by ~94%, the denoising performance did not degrade because overfitting was suppressed effectively. The over-simplified network will also lead to reduced performance because of insufficient network capacity (Supplementary Fig. 2). Thus, using the architecture of 1.0 million trainable parameters is the best choice for practical use. A more comprehensive assessment, including training and inference time, memory consumption and output SNR, is shown in Supplementary Table 1. The lightweight model with ~1.0 million parameters was chosen as the final architecture.

### Data augmentation

The strategy to eliminate overfitting by drastically reducing trainable parameters only works when there is enough training data. If only a small dataset is available, overfitting still occurs even with very small models^70^. To alleviate the data dependency of our method and further eliminate overfitting, we designed 12-fold data augmentation to generate enough training pairs from a small amount of data (Extended Data Fig. 2). Given a low-SNR time-lapse image stack, thousands of 3D training pairs with overlaps will be extracted from the input stack. A training pair includes an input patch and a corresponding target patch. The proportion of temporal overlapping was automatically calculated according to the number of training pairs to be extracted. For each training pair, we first swapped the input and target randomly with a probability of 0.5. Then, we performed six geometric transformations randomly for the training pair, including horizontal flip, vertical flip, left 90° rotation, 180° rotation, right 90° rotation and no transformation. Overall, there were 12 possible forms for each training pair, and they all have the same probability of occurrence, which inflated the training dataset by 12-fold. We investigated the benefit of our data augmentation strategy using synthetic calcium imaging data and found that the data dependency of our method was reduced effectively (Supplementary Fig. 3). A 1,000-frame calcium imaging stack (490 × 490 pixels) is enough to train a model with satisfactory performance. This feature is helpful to alleviate the problem of insufficient training data in fluorescence microscopy. To evaluate the effect of data augmentation on overfitting, we trained one model with data augmentation and another model without data augmentation with the same amount of data for a long training period (35 epochs) and monitored performance after each epoch. The results showed that training with data augmentation could keep the performance stable compared to the rapidly degrading performance without augmentation (Extended Data Fig. 3). The optimal performance was also improved because of augmented training data. Although the combination of model simplification and data augmentation eliminates overfitting, preparing more training data is still the most effective way to improve the denoising performance and avoid overfitting.

### Network architecture, training and inference

**Extended Data Fig. 8:**
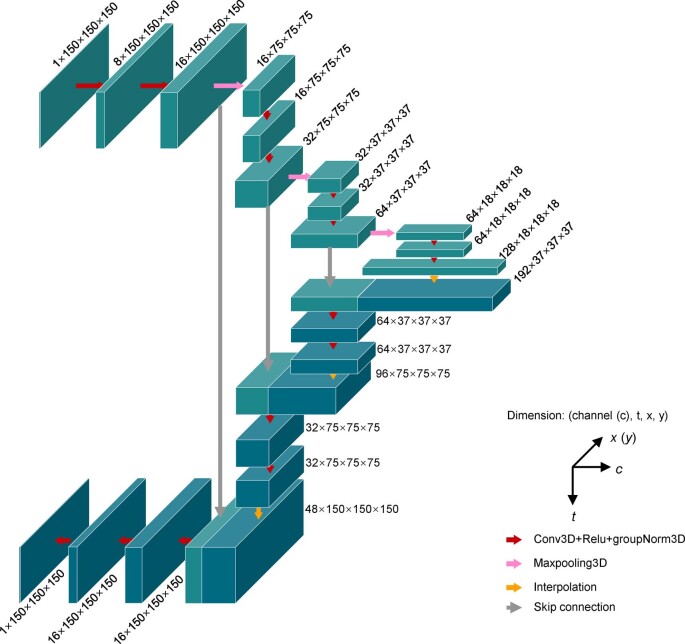
Network architecture. We used simplified 3D U-net^71^ as the network architecture, which is composed of a 3D encoder module, a 3D decoder module, and skip connections from the encoder module to the decoder module. The network architecture was simplified by pruning features in all convolutional layers. The number of trainable parameters was reduced from ~16.3 million (16,315,585) to ~1.0 million (1,020,337) for higher processing speed and less memory consumption.

The network architecture in this research reserves the topology of 3D U-Net^71^ that uses the encoder–decoder architecture in an end-to-end manner. To fully exploit spatiotemporal correlations in fluorescence imaging data, all operations inside the network were implemented in 3D, including convolution, max pooling and interpolation (Extended Data Fig. 8). Compared to our previous architecture^33^, the number of feature maps in each convolutional layer was reduced by fourfold, and the total number of trainable parameters was reduced by 16-fold (1,020,337 compared to 16,315,585), which massively improved the training and inference speed and reduced the memory consumption. For preprocessing, each input stack was subtracted by the average of the whole stack to handle the intensity variation across different samples and imaging platforms. These stacks were partitioned into a specified number of 3D (*x*-*y*-*t*) training pairs. The data augmentation strategy mentioned above would be applied to each training pair. Training was performed using the arithmetic average of an L1-norm loss term and an L2-norm loss term as the loss function. After the input stack flowed through the network, the subtracted average value would be added back after processing. Because the combination of model simplification and data augmentation eliminated overfitting, the model of the last training epoch could be directly selected as the final solution. For denoising of 3D volumetric imaging, the time-lapse stack of each imaging plane was saved as a separate TIFF file. All stacks were used for the training of the network.

The batch size for all experiments was set to the number of GPUs being used. The patch size was set to 150 × 150 × 150 pixels by default. All models were trained using the Adam optimizer^72^ with a learning rate of 5 × 10^−5^, and the exponential decay rates for the first-moment and second-moment estimates were 0.5 and 0.9, respectively. Using our Python code, training with 3,000 pairs of 3D patches for 20 epochs took just 6.2 h on a single GPU (GeForce RTX 3090, Nvidia). The inference process for an image stack composed of 490 × 490 × 300 pixels (partitioned into 75 3D patches) took as few as 8 s. Multi-GPU acceleration has been supported by our Python code. The time consumption of training and inference decreases linearly as the number of GPUs increases.

### Real-time implementation of DeepCAD-RT

To achieve real-time processing during imaging acquisition, we made a program interface to incorporate DeepCAD-RT into our image acquisition software (Scanimage 5.7 (ref. ^73^), Vidrio Technologies). For further acceleration and memory conservation, the inference of DeepCAD-RT was optimally deployed on GPU with TensorRT (Nvidia), a software development kit providing low-latency and high-throughput processing for deep learning applications by executing customized operation automatically for specific GPU and network architecture. Three parallel threads were designed for imaging, data processing and display. The schedule for multithread programming is depicted in Fig. 1c. Specifically, the first thread was used for image acquisition, which waited for a certain number of frames and packaged them into 3D (*x*-*y*-*t*) batches. Adjacent batches had overlapping frames, and half of the overlap would be discarded to avoid artifacts. Then, the second thread got low-SNR images passed by the first thread, processed them and produced denoised frames. Finally, these denoised frames were transferred to the third thread for display. When the imaging process stopped, denoised images would be automatically saved in a user-defined directory. The real-time implementation was programmed in C++ for best hardware interaction and compiled in Matlab (MathWorks), which could be called by any Matlab-based software or script. On a single GPU (GeForce RTX 3090, Nvidia), the real-time implementation achieved more than a 20-fold speed up compared to the original DeepCAD^33^ and had an extremely low memory consumption, as few as 701 MB with float16 precision. The real-time implementation of DeepCAD-RT has been packaged as a free plugin with a user-friendly interface (Extended Data Fig. 1). To transfer pretrained models, scripts were developed to convert PyTorch models to open neural network exchange (ONNX) models and call TensorRT builder to optimize ONNX models for a target GPU, which produced engine files that can be used by TensorRT. The construction of the engine file would eliminate dead computations, fold constants and combine operations to find an optimal schedule for model execution.

### Animal preparation and fluorescence imaging

Multiple animal models (mice, zebrafish and flies) and fluorescence labeling methods (calcium, neutrophils and ATP release) were associated in this research. All experiments involving animals were performed in accordance with the institutional guidelines for animal welfare and have been approved by the Animal Care and Use Committee of Tsinghua University.

#### Mouse preparation and imaging

Adult mice (male or female without randomization or blinding) at 8–16 postnatal weeks were housed in an animal facility (24 °C and 50% humidity) under a reverse light cycle in groups of one to five. All imaging experiments were performed with our two-photon microscopes on head-fixed, awake mice.

For functional imaging of neural activity, we used transgenic mice hybridized between Rasgrf2-2A-dCre mice and Ai148 (TIT2L-GC6f-ICL-tTA2)-D mice expressing Cre-dependent GCaMP6f genetically encoded calcium indicator. Craniotomy surgeries were conducted for chronic two-photon imaging as previously described^33^. Briefly, mice were first anesthetized with 1.5% (by volume in oxygen) isoflurane, and a 6.0-mm-diameter craniotomy was made with a skull drill. After removing the skull piece, a coverslip was implanted on the craniotomy region, and a titanium headpost was then cemented to the skull for head fixation. After the surgery, 0.25 mg per gram (body weight) trimethoprim was injected intraperitoneally to induce the expression of GCaMP6f in layer 2/layer 3 cortical neurons across the whole brain. After inflammation was gone and the cranial window became clear (~2 weeks after surgery), mice were head-fixed on a customized holder with a 3D-printed plastic tube to restrict the mouse body. The holder was mounted on a high-precision, three-axis motorized stage (M-VP-25XA-XYZL, Newport) for sample translation. In vivo calcium imaging (30-Hz single-plane imaging) was performed on awake mice without anesthesia. The imaging of dendritic spines in L1 (20–60 μm below the brain surface) required adequate spatial sampling rate that was achieved by using large zoom factors.

For time-lapse imaging of neutrophil migration, we first performed craniotomies on wild-type mice (C57BL/6J) following the procedures described above. Acute brain injury caused by craniotomy induce immune responses in the brain. After surgery, neutrophils and blood vessels were simultaneously labeled by injecting 10 μg of red (Alexa Fluor 555 conjugate) wheat germ agglutinin (WGA) dye (W32464, Thermo Fisher Scientific) and 2 μg of green-fluorescence-conjugated Ly-6G/Ly-6C antibody (53-5931-82, eBioscience) intravenously. The two dyes were dissolved and diluted in 200 μl of 1× PBS. To avoid the potential influence of anesthesia on immune responses, in vivo two-photon imaging was performed in the mouse brain after the mouse was fully awake (~20 min after injection). Imaging experiments should be finished as soon as possible because these dyes are degradable in the mouse body. Empirically, the whole imaging session should take no longer than 5 h. Volumetric imaging was implemented by scanning the objective axially with the piezoelectric actuator. The frame rate of single-plane imaging was 30 Hz, and the volume rate of 3D imaging was 2 Hz (15 imaging planes). The whole 3D imaging session lasted ~20 min. For each 3D volume, the flyback frame acquired while the piezoelectric actuator was quickly returning from the bottom plane to the top plane should be discarded. Images of the green channel and the red channel were captured simultaneously and were separated by postprocessing.

For functional imaging of ATP dynamics, wild-type mice (C57BL/6J) were anesthetized with intraperitoneally injected Avertin (500 mg per kilogram (body weight), Sigma-Aldrich). A cranial window was opened on the visual cortex, and 400–500 nl of adeno-associated virus (AAV2/9-GfaABC1D-ATP1.0, packaged at Vigene Biosciences) was injected (anterior–posterior: −2.2 mm relative to bregma, medial–lateral: 2.0 mm relative to bregma and dorsal–ventral: 0.5 mm below the dura, at an angle of 30°) using a microsyringe pump (Nanoliter 2000 injector, World Precision Instruments) to express GRAB_ATP1.0_ (ref. ^34^) in cortical astrocytes. A 4 mm × 4 mm square coverslip was implanted to replace the skull. After ~3 weeks of recovery and virus expression, two-photon imaging was performed to record ATP release events in the mouse cortex. Before imaging, brain injury was induced by ablating the tissue with a stationary laser focus (200 mW) for 5 s. The injury site was located at the center of the 3D imaging volume. Single-plane images were recorded at the plane 20 μm above the injury site. The frame rate of single-plane imaging was 30 Hz, and the volume rate of 3D imaging was 1 Hz (30 imaging planes). The flyback frame of each volume should be discarded. Only signals from the green channel were recorded, and the whole 3D imaging session lasted 60 min.

#### Zebrafish preparation and imaging

Transgenic zebrafish (*Danio rerio*) larvae expressing pan-neuronal GCaMP6s calcium indicator (Tg(HuC:GCaMP6s)) were housed in culture dishes at 28.5 °C in Holtfreter’s solution (59 mM NaCl, 0.67 mM KCl, 0.76 mM CaCl_2_ and 2.4 mM NaHCO_3_). At 4–6 d after fertilization, zebrafish larvae were separated and restricted in a small drop of 1.0% low-melting-point agarose (Sigma-Aldrich) and mounted on a microscope slide for imaging. A fine-bristle brush was used to adjust the posture of the larvae to keep the dorsal side up before the agarose solidified. After fixation, the larvae were placed under the objective, and Holtfreter’s solution was used as the immersion medium of the objective. Before image acquisition started, we previewed the image and rotated the microscope slide manually to keep the larva horizontal or vertical in the FOV. Two-photon calcium imaging of spontaneous neural activity was performed on the larvae at 26–27 °C without anesthesia or motion paralysis. All experiments were single-plane imaging, and the frame rate was 30 Hz for 512 × 512 pixels. Both large neuronal populations across multiple brain regions and small neuronal subsets localized in the optic tectum were imaged using different zoom factors.

#### *Drosophila* preparation and imaging

Flies were raised on standard cornmeal medium with a 12-h light/12-h dark cycle at 25 °C. Transgenic flies UAS-GCaMP7f were crossed with OK107-Gal4 to drive the expression of the GCaMP7f^25^ calcium indicator in essentially all Kenyon cells. All experiments were conducted on female F1 heterozygotes from this cross. Flies at 5 d after eclosion were anesthetized on ice and mounted in a 3D-printed plastic disk that allowed free movement of the legs, as previously reported^74^. The posterior head capsule was opened using sharp forceps (5SF, Dumont) at room temperature in carbonated (95% O_2_, 5% CO_2_) buffer solution (103 mM NaCl, 3 mM KCl, 5mM N-Tris, 10 mM trehalose, 10 mM glucose, 7 mM sucrose, 26 mM NaHCO_3_, 1 mM NaH_2_PO_4_, 1.5 mM CaCl_2_ and 4 mM MgCl_2_) with a pH of 7.3 and an osmolarity of 275 mosM. After that, the air sacks and tracheas were also removed. Brain movement was minimized by adding UV glue around the proboscis and removing the M16 muscle^40,75^. After preparation, flies were placed under the objective for two-photon imaging of calcium transients in the mushroom body. To enhance neural activity, 4-methylcyclohexanol and 3-octanol diluted 1:1,000 in mineral oil were used as odors. Flies were randomly given the two odors for 5 s every 10 s using a custom-made air pump. All experiments were single-plane imaging experiments at 30 Hz with 512 × 512 pixels.

### Generation of synthetic calcium imaging data

We used synthetic calcium imaging data (simulated time-lapse image sequences) for quantitative evaluations of our method and for comparisons with DeepInterpolation^32^. Our simulation pipeline consisted of synthesizing noise-free calcium imaging videos (ground truth) and adding different levels of mixed Poisson–Gaussian noise^22,33^. To generate noise-free calcium imaging data, we adopted in silico NAOMi, a simulation method to create realistic calcium imaging datasets for assessing two-photon microscopy methods^36^. The parameters of our simulation are listed in Supplementary Table 2. Those not mentioned all used default values. Simulated data had very similar spatiotemporal features to experimentally obtained data, including neuronal anatomy (cell bodies, neuropils, dendrites and so on), neural activity and blood vessels. For noise simulation, we first performed Poisson sampling on noise-free images to simulate the content-dependent Poisson noise. We then added content-independent Gaussian noise to these data. Poisson noise was set as the dominant noise source. Different imaging SNRs were simulated by different relative photon numbers that changed the intensity of input noise-free images (Supplementary Fig. 1).

### Neutrophil segmentation

Four types of data were involved in this experiment, that is, raw data (low-SNR), high-SNR (tenfold fluorescence photons) data, denoised raw data and denoised high-SNR data. Ten representative images with relatively sparse cells were selected from the dataset of single-plane neutrophil imaging for semantic segmentation. To obtain ground-truth segmentation masks, five human experts were recruited to annotate all neutrophils in each denoised high-SNR image using the ROI Manager toolbox of Fiji. The final ground-truth masks were determined by majority voting. Neutrophil segmentation was conducted using Cellpose^46^ and Stardist^47^, two CNN-based generalist algorithms for cellular segmentation. For both methods, default parameters and pretrained models were used without additional training. Segmentation performance was quantitatively evaluated with the IoU score^76^ defined as

$${{{\mathrm{IoU}}}} = \frac{{{{{\mathrm{A}}}} \cap {{{\mathrm{B}}}}}}{{{{{\mathrm{A}}}} \cup {{{\mathrm{B}}}}}},$$

where A is the mask segmented by algorithms and B is the ground truth. Statistical analysis and representative results are summarized in Extended Data Fig. 7.

### Three-dimensional visualization

For volumetric imaging of neutrophil migration and ATP release, we performed 3D visualization to reveal the spatiotemporal patterns of biological dynamics. Imaris 9.0 (Oxford Instruments) was used for the visualization of all volumetric imaging data. Both the original low-SNR data and denoised data were imported into Imaris, rendered with pseudocolor and 3D reconstructed using the maximum intensity projection mode. The brightness of data before and after denoising was adjusted to make them have a similar visual effect. The contrast of low-SNR data was fine-tuned to show underlying signals as clearly as possible. All values for gamma correction were set to one. The red channel (blood vessels) of neutrophil migration was averaged by multiple frames to improve its SNR and merged with the green channel. Cross-talk signals out of the blood vessel were manually suppressed with Fiji. Animations were generated by automatically interpolating intermediate frames between selected keyframes.

### Annotation of ATP release events

The whole annotation pipeline was implemented on the denoised data (Supplementary Fig. 8). The spatial shape of each ATP release event could be modeled as an ellipsoid. To obtain the center position and peak time of each event throughout the whole imaging session, we manually annotated them by adding measurement points in Imaris. All spatial and temporal coordinates were exported from the software after annotation. Events at the edge of the volume were excluded because only a part of them appeared in the FOV. Based on these annotated coordinates, intensity profiles along all three dimensions of each event were extracted from denoised stacks with a custom Matlab (MathWorks) script. Gaussian fitting was performed for all intensity profiles to reduce the influence of background fluctuations. All fitted Gaussian curves were then deconvolved with the system point spread function using a standard Richardson–Lucy algorithm^77,78^. This step eliminated the influence of limited and anisotropic spatial resolution. The diameter of these ATP release events could be extracted in each dimension, which was defined as the FWHM of deconvolved Gaussian curves. The ellipticity of release events was defined as

$${{{\mathrm{ellipticity}}}} = \frac{{a - b}}{a},$$

where *a* is the major axis of the ellipse, and *b* is the minor axis of the ellipse. Ellipticity was calculated for each 3D release event in all three orthogonal coordinate planes (*x*-*y*, *y*-*z* and *x*-*z*).

### Method comparison

Four baseline methods are included in the comparison. Synthetic calcium imaging images (6,000 frames, 30 Hz frame rate) were used for the training and testing of all methods. For each method, a specified model was trained for each SNR level. The supervised baseline was obtained with a larger 3D U-Net (4.1 million trainable parameters) trained in a supervised manner. All hyperparameters were kept the same with DeepCAD-RT. DeepInterpolation was implemented with the companion code of relevant papers^32^, and two kinds of DeepInterpolation models were trained using default hyperparameters. The first model was trained from scratch. The other model was fine-tuned based on a pretrained model (pretrained with 225,000 two-photon images of the Ai93 reporter line) by presenting the training data only once according to the DeepInterpolation paper. Noise2Void^37^ models were trained for 50 epochs with 64 × 64 patch size and 128 batch size. HDN is the upgraded version of DivNoising^79^ with state-of-the-art performance. Because no calibration data are available, the noise models of HDN were bootstrapped from the noisy data, and the conditional distributions were estimated from paired noisy images and pseudo-ground truth (obtained from Noise2Void). The noise models were trained for 10,000 epochs with a batch size of 250,000 and 0.01 learning rate. The final HDN model of each SNR was trained for 150 epochs, and the best training epoch was selected by evaluating the output SNR of the first 10 frames. The minimum mean square error estimate of each frame was obtained by averaging 100 denoised samples. All hyperparameters not mentioned here were set as default values.

### Performance metrics

To quantitatively evaluate the performance of our method, both synthetic data and experimentally obtained data were used. For synthetic calcium imaging data, ground-truth images were available, and SNR was calculated to quantify the denoising performance. SNR was defined as the logarithmic form

$${\mathrm{SNR}} = 10 \cdot \log _{10}\frac{{\left\| y \right\|_2^2}}{{\left\| {x - y} \right\|_2^2}},$$

where *x* is the denoised data, and *y* is the ground truth. For experimentally obtained data, synchronized high-SNR data with tenfold photons acquired with our system were used as the reference of underlying signals. Pearson correlation coefficient (*R*) was used as the performance metric, which is formulated as

$$R = \frac{{{{{\mathrm{E}}}}\left[ {(x - \mu _x)(y - \mu _y)} \right]}}{{\sigma _x\sigma _y}},$$

where *x* and *y* are the denoised data and corresponding high-SNR data, respectively; *μ*_*x*_ and *μ*_*y*_ are the mean values of *x* and *y*; and *σ*_*x*_ and *σ*_*y*_ are the standard deviations. The operator E represents arithmetically averaging. Pearson correlation was used for both images and fluorescence traces. All performance metrics were implemented with custom Matlab scripts and built-in functions.

### Statistics and reproducibility

Sample sizes and statistics are reported in the figure legends and text for each experiment. All box plots were plotted in the format of standard Tukey box and whisker plots. The box indicates the lower and upper quartiles, while the line in the box shows the median. The lower whisker represents the first data point greater than the lower quartile minus 1.5× the interquartile range. Similarly, the upper whisker represents the last data point less than the upper quartile plus 1.5× the interquartile range. Outliers were plotted in small black dots. For the comparison of images and fluorescence traces before and after denoising, a one-sided paired *t*-test was performed, and *P* values are indicated with asterisks. Representative frames were demonstrated in the figures, and similar results were achieved on more than 1,500 frames for all experiments.

### Reporting summary

Further information on research design is available in the Nature Research Reporting Summary linked to this article.

## Online content

Any methods, additional references, Nature Research reporting summaries, source data, extended data, supplementary information, acknowledgements, peer review information; details of author contributions and competing interests; and statements of data and code availability are available at 10.1038/s41587-022-01450-8.

## Supplementary information

Supplementary Information Supplementary Figs. 1–8 and Tables 1 and 2.

Reporting Summary

Supplementary Video 1 Demonstrating real-time denoising on a two-photon microscope using DeepCAD-RT.

Supplementary Video 2 DeepCAD-RT enhances the in vivo recording of calcium transients in dendritic spines.

Supplementary Video 3 DeepCAD-RT massively improves the imaging SNR of neuronal population recordings in the zebrafish brain.

Supplementary Video 4 DeepCAD-RT massively improves the imaging SNR of neuronal population recordings across multiple brain regions in the zebrafish brain.

Supplementary Video 5 DeepCAD-RT enhances neuronal population imaging of *Drosophila* mushroom bodies.

Supplementary Video 6 Denoising performance of DeepCAD-RT on two-photon imaging of neutrophils in the mouse brain.

Supplementary Video 7 DeepCAD-RT facilitates high-SNR observations of retraction fiber dynamics during neutrophil migration.

Supplementary Video 8 DeepCAD-RT reveals the 3D migration of neutrophils in vivo after acute brain injury.

Supplementary Video 9 Denoising performance of DeepCAD-RT on a recently developed genetically encoded ATP sensor.

Supplementary Video 10 DeepCAD-RT reveals the ATP dynamics of astrocytes in 3D after laser-induced brain injury.

## Data Availability

We have no restriction on data availability. All source data (~250 GB), including synthetic calcium imaging data, experimental recordings of calcium dynamics, neutrophil migration and cortical ATP release, have been archived and made publicly available at https://cabooster.github.io/DeepCAD-RT/Datasets/. Source data are provided with this paper.

## Source data

Source Data Fig. 2 Statistical source data.
Source Data Fig. 3 Statistical source data.
Source Data Fig. 4 Statistical source data.
Source Data Fig. 5 Statistical source data.
Source Data Extended Data Fig. 6 Statistical source data.
Source Data Extended Data Fig. 7 Statistical source data.

## Code availability

All relevant resources are readily accessible on our GitHub page at https://cabooster.github.io/DeepCAD-RT/. The source PyTorch code, demo notebooks (in Jupyter Notebook and Google Colab) and the code for real-time implementation can be found at https://github.com/cabooster/DeepCAD-RT/. A detailed tutorial for all codes has been provided at https://cabooster.github.io/DeepCAD-RT/Tutorial/.
