# Supplementary material for: Real-time denoising enables high-sensitivity fluorescence time-lapse imaging beyond the shot-noise limit
Source: Nat Biotechnol. 2022 Sep 26;41(2):282–92. doi: 10.1038/s41587-022-01450-8 (PMC9931589; doi:10.1038/s41587-022-01450-8)
Supplement: Supplementary file 1 — Supplementary Figs. 1–8 and Tables 1 and 2. [file 41587_2022_1450_MOESM1_ESM.pdf]

nature portfolio

https://doi.org/10.1038/s41587-022-01450-8

Supplementary information

Real-time denoising enables high-sensitivity fluorescence time-lapse imaging beyond the shot-noise limit

In the format provided by the authors and unedited

Supplementary Information

Real-time denoising enables high-sensitivity fluorescence time-lapse imaging beyond the shot-noise limit

Xinyang Li[1,2,3,4†], Yixin Li[3,5†], Yiliang Zhou[1,3], Jiamin Wu[1,3,6,7], Zhifeng Zhao[1,3], Jiaqi Fan[2,3,8], Fei Deng[9,10], Zhaofa Wu[9,10], Guihua Xiao[1,3], Jing He[1,3], Yuanlong Zhang[1,3], Guoxun Zhang[1,3], Xiaowan Hu[2], Xingye Chen[1,3], Yi Zhang[1,3], Hui Qiao[1,3,6], Hao Xie[1,3], Yulong Li[9,10], Haoqian Wang[2,3*], Lu Fang[3,8*] & Qionghai Dai[1,3,6,7*]

[1]Department of Automation, Tsinghua University, Beijing 100084, China.

[2]Tsinghua Shenzhen International Graduate School, Tsinghua University, Shenzhen, 518055, China.

[3]Institute for Brain and Cognitive Sciences, Tsinghua University, Beijing 100084, China.

[4]Hangzhou Zhuoxi Institute of Brain and Intelligence, Hangzhou, 311100, China.

[5]School of Information and Technology, Fudan University, Shanghai 200433, China.

[6]Beijing Key Laboratory of Multi-dimension & Multi-scale Computational Photography (MMCP), Tsinghua University, Beijing 100084, China.

[7]IDG/McGovern Institute for Brain Research, Tsinghua University, Beijing, China.

[8]Department of Electronic Engineering, Tsinghua University, Beijing 100084, China.

[9]State Key Laboratory of Membrane Biology, Peking University School of Life Sciences, Beijing 100871, China.

[10]PKU-IDG/McGovern Institute for Brain Research, Beijing 100871, China.

[†]These authors contributed equally to this work.

[*]Correspondence: wanghaoqian@tsinghua.edu.cn; fanglu@tsinghua.edu.cn; qhdai@tsinghua.edu.cn

CONTENT

I. Supplementary Figures

**Supplementary Figure 1**	*In silico* simulation of calcium imaging data.
**Supplementary Figure 2**	Evaluating the performance of different model complexity.
**Supplementary Figure 3**	Evaluating the data dependency of DeepCAD-RT.
**Supplementary Figure 4**	Comparing DeepCAD-RT with other methods at different noise levels.
**Supplementary Figure 5**	Representative images for method comparison.
**Supplementary Figure 6**	Performance comparison between DeepCAD-RT and DeepInterpolation on neutrophil imaging data.
**Supplementary Figure 7**	System point spread function (PSF).
**Supplementary Figure 8**	ATP annotation pipeline.

II. Supplementary Tables

**Supplementary Table 1**	Comparison of different model complexity.
**Supplementary Table 2**	Parameters for the simulation of calcium imaging data.

III. Supplementary Videos

**Supplementary Video 1**	Demonstrating real-time denoising on a two-photon microscope using DeepCAD-RT.
**Supplementary Video 2**	DeepCAD-RT enhances the *in vivo* recording of calcium transients in dendritic spines.
**Supplementary Video 3**	DeepCAD-RT massively improves the imaging SNR of neuronal population recordings in the zebrafish brain.
**Supplementary Video 4**	DeepCAD-RT massively improves the imaging SNR of neuronal population recordings across multiple brain regions in the zebrafish brain.
**Supplementary Video 5**	DeepCAD-RT enhances the neuronal population imaging of Drosophila mushroom body.
**Supplementary Video 6**	Denoising performance of DeepCAD-RT on two-photon imaging of neutrophils in the mouse brain.
**Supplementary Video 7**	DeepCAD-RT facilitates high-SNR observations of retraction fiber dynamics during neutrophil migration.
**Supplementary Video 8**	DeepCAD-RT reveals the 3D migration of neutrophils *in vivo* after acute brain injury.
**Supplementary Video 9**	Denoising performance of DeepCAD-RT on a recently developed genetically encoded ATP (adenosine 5'-triphosphate) sensor.
**Supplementary Video 10**	DeepCAD-RT reveals the ATP (adenosine 5'-triphosphate) dynamics of astrocytes in 3D after laser-induced brain injury.

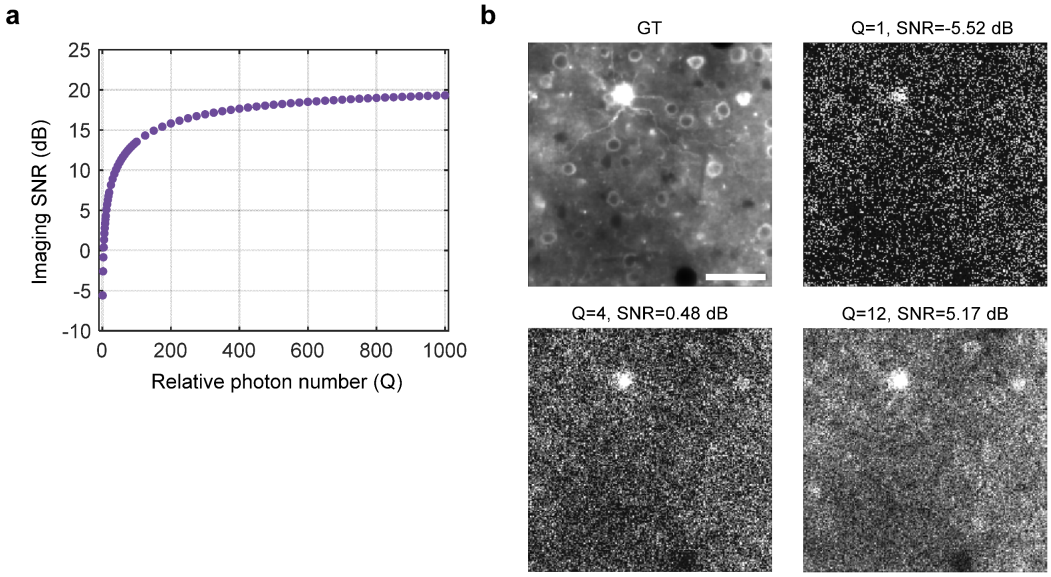

Supplementary Figure 1

In silico simulation of calcium imaging data.

Noise-free two-photon calcium imaging videos were simulated with *in silico* Neural Anatomy and Optical Microscopy (NAOMi)[1]. Different levels of Mixed Poisson-Gaussian (MPG) noise[2, 3] were added subsequently. Noise-free images were used as the ground truth for quantitative evaluations of the denoising performance. **a,** The relation between imaging SNR and relative photon number (Q). **b,** Representative images of different imaging SNRs and corresponding ground truth (GT). Scale bar, 50 μm.

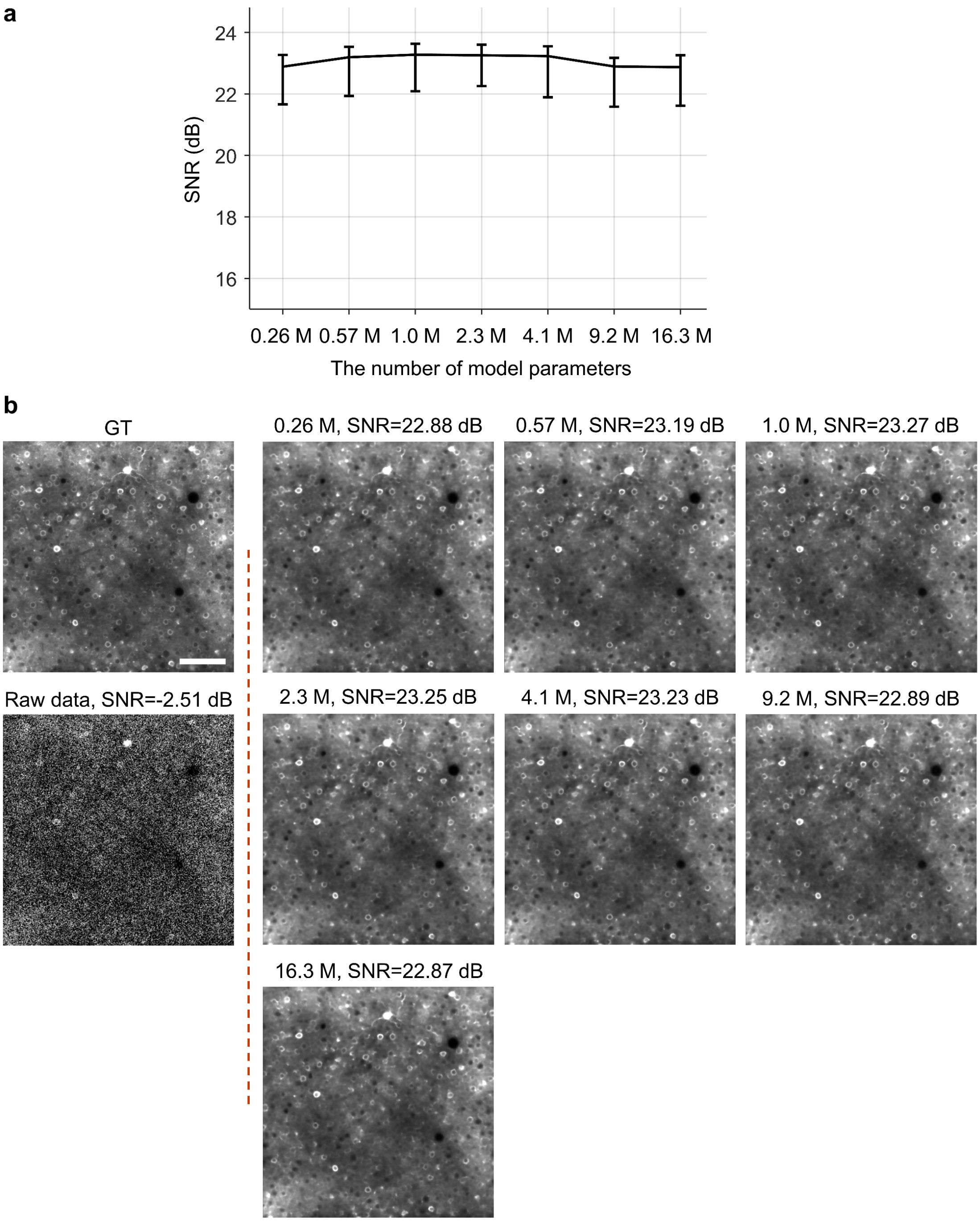

Supplementary Figure 2

Evaluating the performance of different model complexity.

Simulated calcium imaging data (6000 frames, 30 Hz frame rate, SNR=-2.51 dB) were used in this experiment for quantitative evaluation. The best training epoch was selected by validation. **a**, The relation between denoising performance (output SNR) and the number of model parameters. The line shows mean values and error bars represent the minimum and maximum values. **b**, Example ground truth (GT) images, raw data before denoising, and images after denoising. Scale bar, 100 μm.

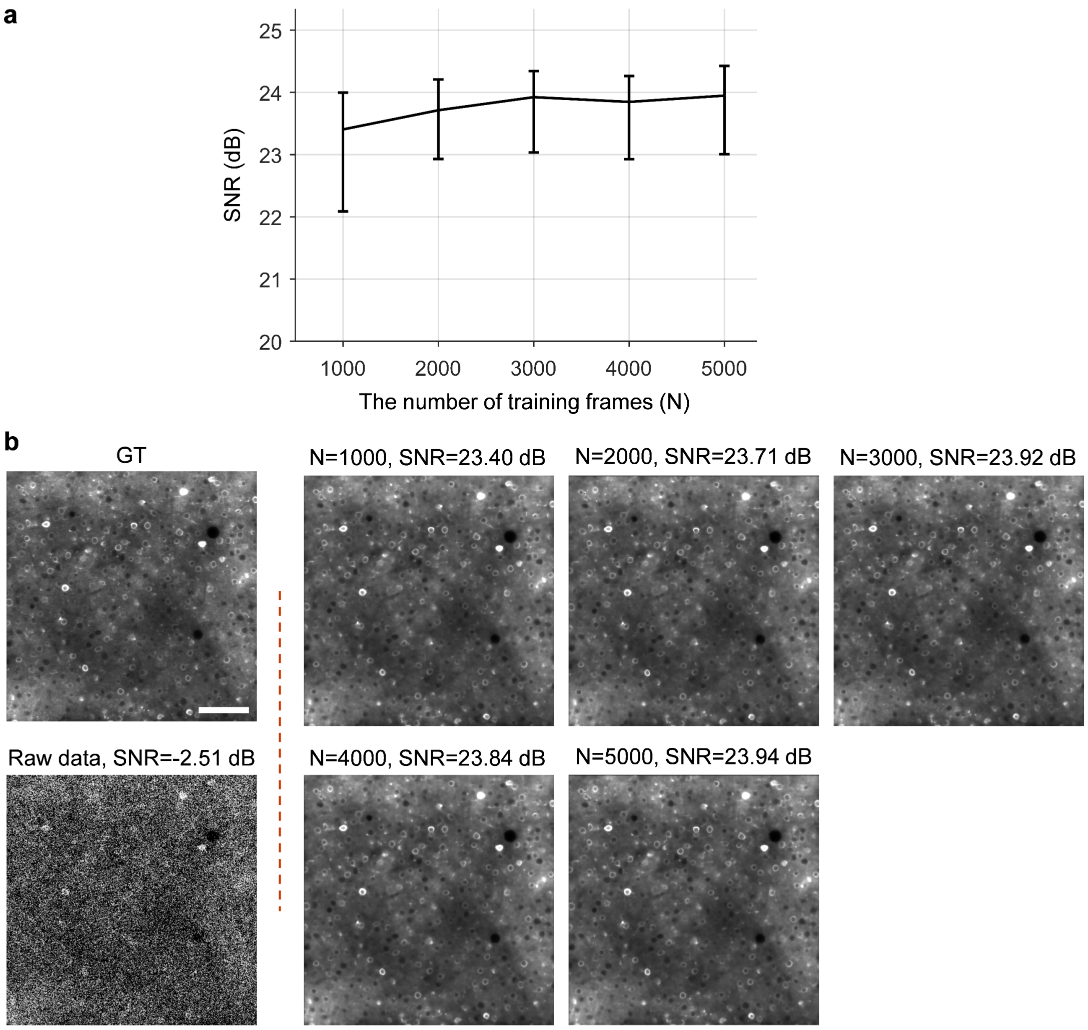

Supplementary Figure 3

Evaluating the data dependency of DeepCAD-RT.

Simulated calcium imaging data (30 Hz frame rate, SNR=-2.51 dB) were used in this experiment for quantitative evaluation. The network architecture has been simplified (~1.0 million trainable parameters) and data augmentation was applied. Each model was trained for 20 epochs and the last epoch was used for comparison. **a**, The relation between denoising performance (output SNR) and the number of training frames (N). The line shows mean values and error bars represent the minimum and maximum values. **b**, Example ground truth (GT) images, raw data before denoising, and images after denoising. Scale bar, 100 μm.

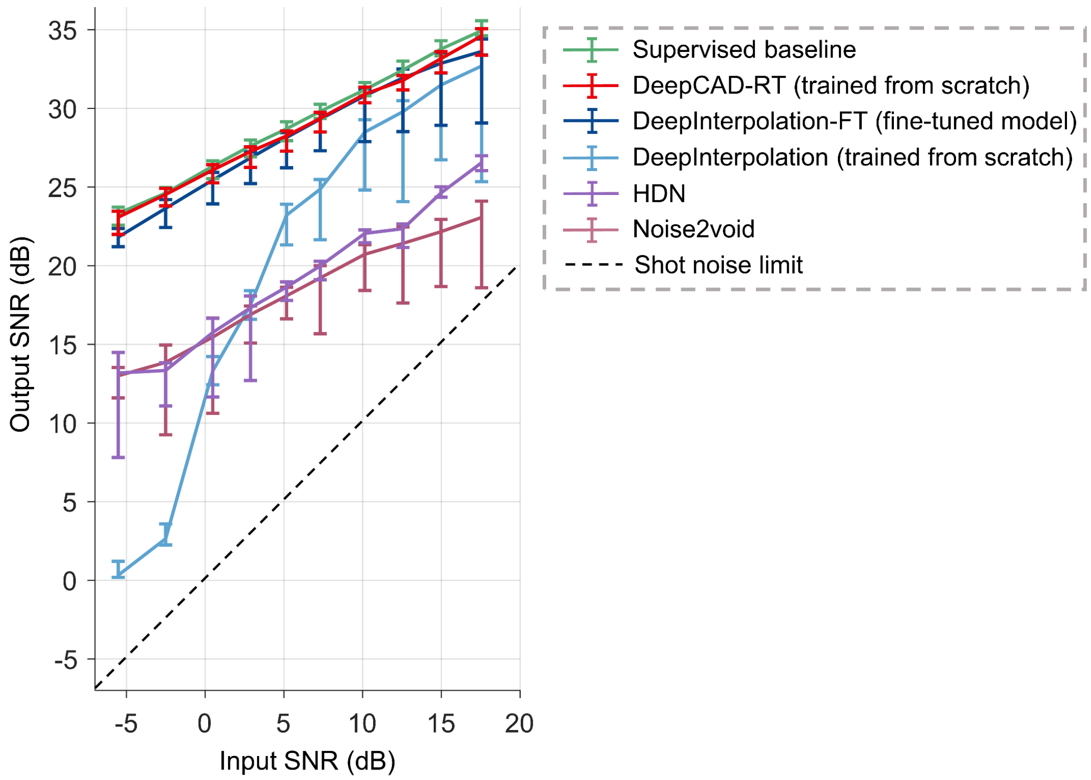

Supplementary Figure 4

Comparing DeepCAD-RT with other methods at different noise levels.

The relationship between the input SNR and output SNR of different denoising methods. Simulated calcium imaging data (6000 frames, 30 Hz frame rate) were used for the training of all methods. DeepCAD-RT (simplified network architecture with ~1.0 million trainable parameters) was trained from scratch for 20 epochs with data augmentation. DeepInterpolation was implemented with the companion code of relevant papers[4] and two kinds of DeepInterpolation models were trained. The first one was trained from scratch. The other model was fine-tuned based on a pre-trained model by presenting the training data only once according to the DeepInterpolation paper. The supervised baseline was obtained with a 3D-Unet (4.1 M trainable parameters) trained in a supervised manner. Noise2Void[5] and Hierarchical DivNoising (HDN)[6] are 2D methods and were implemented with the companion code of relevant papers. Each Noise2Void model was trained for 50 epochs. Each HDN model was trained for 150 epochs and the minimum mean square error (MMSE) estimate of each frame was obtained by averaging 100 denoised samples. For each method, a specified model was trained for each SNR level. Lines represent mean values and error bars represent the minimum and maximum values. The black dashed line represents the shot-noise limit (*i.e.*, the input SNR is equal to the output SNR).

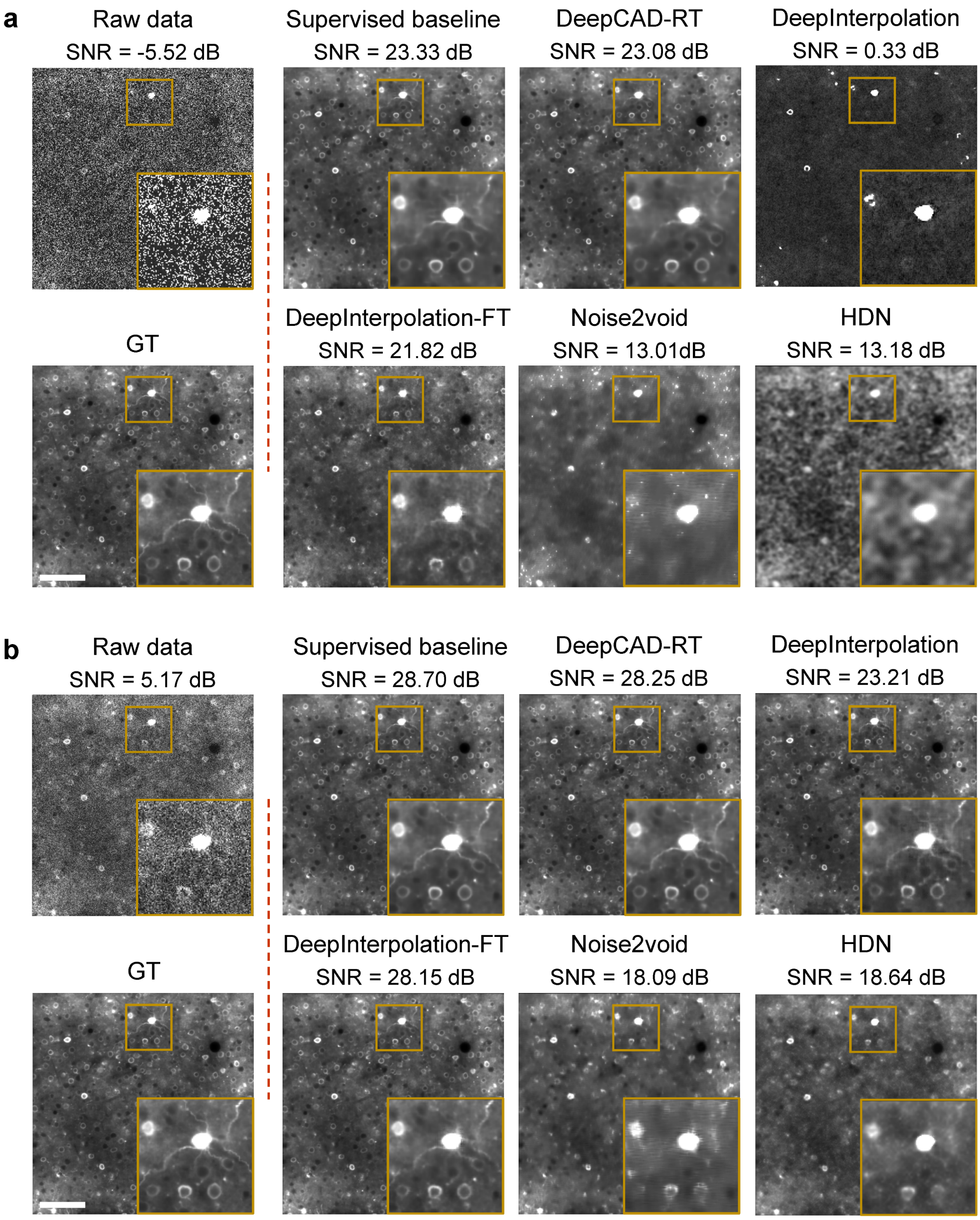

Supplementary Figure 5

Representative images for method comparison.

Representative images for comparison of different methods presented in Supplementary Figure 4. **a**, Results when the input SNR is -5.52 dB. Scale bar, 100 μm. **b**, Results when the input SNR is 5.17 dB. Scale bar, 100 μm. Magnified views of yellow boxed regions are shown at the right bottom of each image.

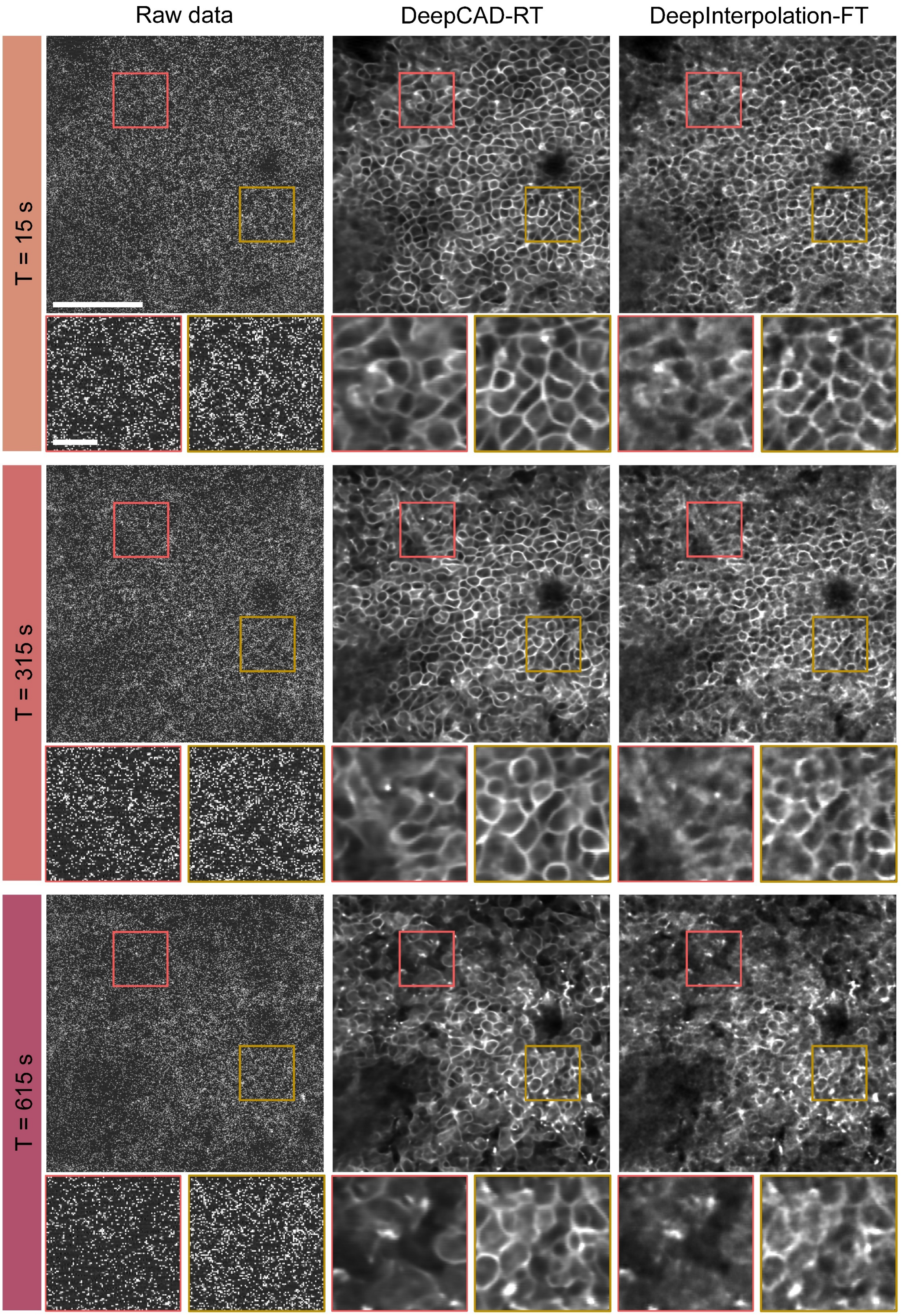
Raw data
DeepCAD-RT
DeepInterpolation-FT
T = 15 s
T = 315 s
T = 615 s

Supplementary Figure 6

Performance comparison between DeepCAD-RT and DeepInterpolation on neutrophil imaging data.

Neutrophil imaging data (2000 frames, 2 Hz frame rate) were used to train our method and fine-tune DeepInterpolation (DeepInterpolation-FT). Three frames at different moments are demonstrated here and each row shows one frame. Magnified views of boxed regions are shown under each image. Scale bar, 50 μm for large field-of-views and 10 μm for magnified views.

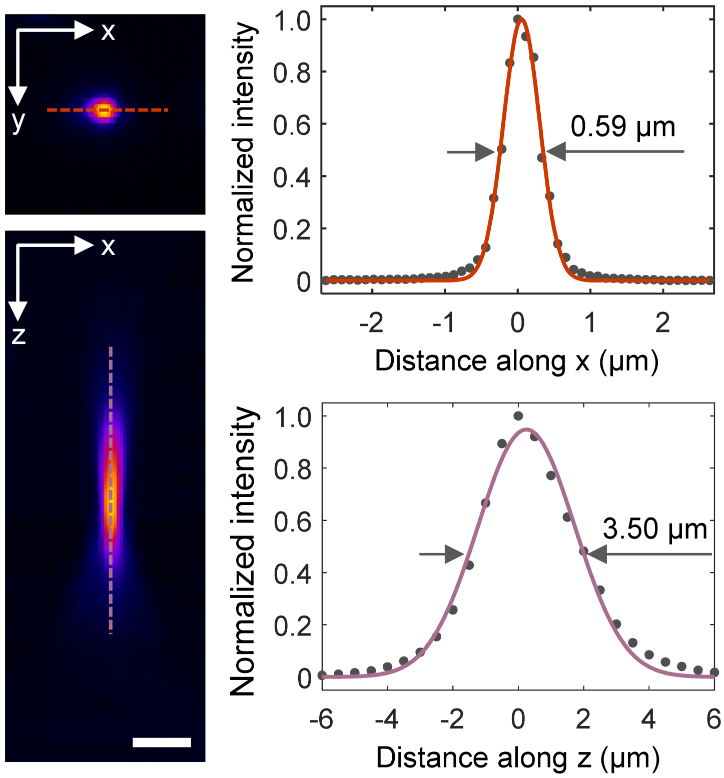

Supplementary Figure 7

System point spread function (PSF).

The spatial resolution of our imaging system was calibrated by imaging 0.2-μm fluorescent beads. The lateral (XY) and axial (XZ) projections of the system PSF are shown on the left. Normalized intensity profiles of the dashed lines are shown on the right. Solid lines are gaussian fitted curves. The lateral resolution (defined in FWHM) of our system is 0.59 μm and the axial resolution is 3.50 μm. Scale bar, 2 μm.

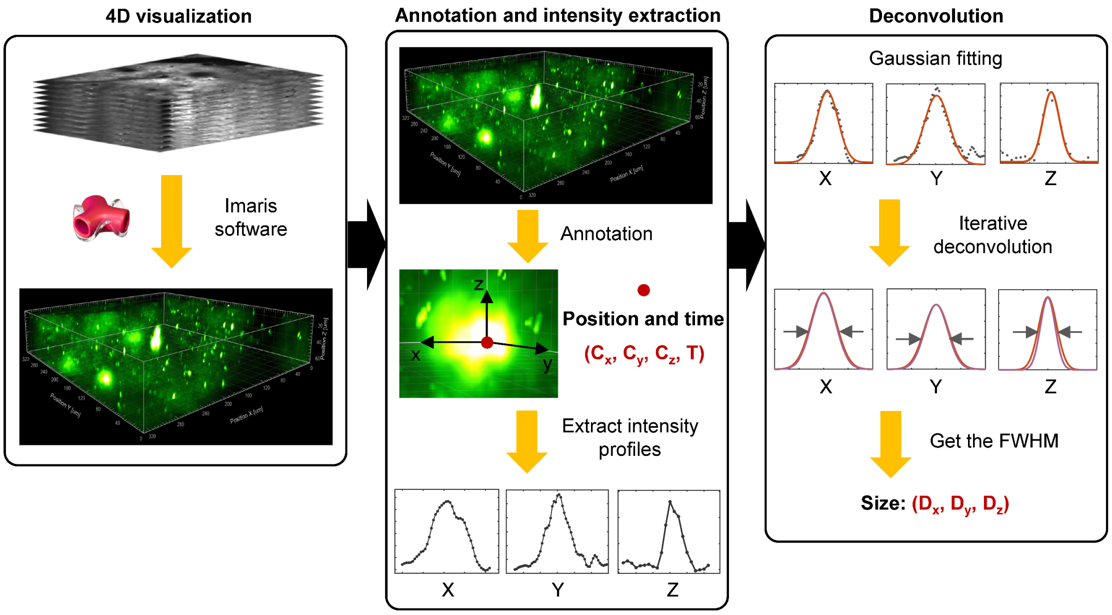

Supplementary Figure 8

ATP annotation pipeline.

First, low-SNR recordings (4D, xyz-t) were denoised with DeepCAD-RT. Denoised data were visualization in Imaris software. Second, all ATP-release events during the whole imaging session were manually annotated to obtain their position and time. Then, intensity profiles along all three dimensions of each event were extracted from the denoised data. Finally, Gaussian fitting was performed and fitted curves were deconvoluted with the Richardson–Lucy algorithm to eliminate the influence of limited and anisotropic spatial resolution. The full width at half maximum (FWHM) of each deconvoluted curve was extracted as the size of the event.

Supplementary Table 1

Comparison of different model complexity.

We compared the memory cost, time consumption, and denoising performance of different model complexity using simulated calcium imaging data with clean images for better quantification. All hyper-parameters were kept unchanged except the total number of model parameters. Specifically, all models were trained on 6000 pairs of 150×150×150 (x×y×t) 3D image patches for 10 epochs with one batch size. For inference, a 490×490×300 calcium imaging sequence was split into 75 150×150×150 3D patches and then fed into each model with one batch size. The GPU for training and inference was Nvidia GeForce RTX 3090.

The number of model parameters	Model file size (MB)[a]	Training memory (GB)	Training time (h)	Inference memory (GB)	Inference time (s)[b]	Output SNR (dB)[c]
16,315,585 (16.3 M)	62.3	13.0	35.3	18.7	53	22.87
9,178,129 (9.2 M)	35.0	10.1	25.9	14.4	40	22.89
4,079,713 (4.1 M)	15.6	7.2	13.1	10	16	23.23
2,295,145 (2.3 M)	8.8	5.7	9.9	7.9	12	23.25
1,020,337 (1.0 M)	3.9	4.3	6.2	5.7	8	23.27
574,093 (0.57 M)	2.2	4.0	4.9	4.7	6	23.19
255,289 (0.26 M)	1.0	2.9	3.4	3.6	4	22.88

a. The size of each model file (*.pth).

b. Since the simulated frame rate is 30 Hz, the imaging time for 300 frames is about 10 s.

c. The SNR of the input noisy data is -2.51 dB. The output SNR of each model was measured at the optimal training epoch validated with the ground-truth data.

Supplementary Table 2

Parameters for the simulation of calcium imaging data

We used NAOMi[1] to generate realistic two-photon calcium imaging data. These simulated noise-free videos were used as the ground truth for comparisons of different denoising methods and evaluations of our method. All simulated data were single-plane time-lapse image sequence. The key simulation parameters are as follows. Those not mentioned in the table all used default values.

**Physiological parameters**		**Imaging parameters**	
Sample volume	500×500×50 μm	FOV[a]	500×500 μm
Average neuron radius	5.9 μm	Pixel size	1.02 μm
Number of neurons	1250	Image size	490×490 pixels
Average firing rate	0.25	Frame rate	30 Hz
Vasculature simulation	ON	Imaging depth	200 μm
Background dendrites	ON	Excitation NA[b]	0.6
Calcium indicator	GCaMP6	Detection NA	0.8
Fluorophore concentration	10 μM	Excitation power	50 mW
**Laser source**		Number of frames	6000
Wavelength	920 nm	Objective focal length	4.5 mm
Repetition frequency	80 MHz	Brain motion	OFF
Pulse width	150 fs	PSF[c] type	Gaussian

a. FOV: field-of-view of the microscope.

b. NA: Objective numerical aperture.

c. PSF: point spread function of the optical system.

References

1. Song, A., Gauthier, J.L., Pillow, J.W., Tank, D.W. & Charles, A.S. Neural anatomy and optical microscopy (NAOMi) simulation for evaluating calcium imaging methods. J. *Neurosci. Methods* **358**, 109173 (2021).
2. Meiniel, W., Olivo-Marin, J.C. & Angelini, E.D. Denoising of microscopy images: A review of the state-of-the-art, and a new sparsity-based method. *IEEE Trans. Image Process.* 27, 3842-3856 (2018).
3. Li, X. et al. Reinforcing neuron extraction and spike inference in calcium imaging using deep self-supervised denoising. *Nat. Methods* **18**, 1395-1400 (2021).
4. Lecoq, J. et al. Removing independent noise in systems neuroscience data using DeepInterpolation. *Nat. Methods* **18**, 1401-1408 (2021).
5. Krull, A., Buchholz, T.-O. & Jug, F. Noise2Void-learning denoising from single noisy images. in *Proc. IEEE Conference on Computer Vision and Pattern Recognition* 2129-2137 (2019).
6. Prakash, M., Delbracio, M., Milanfar, P. & Jug, F. Interpretable unsupervised diversity denoising and artefact removal. in *International Conference on Learning Representations* (2022).
