# Supplementary material for: Real-time denoising enables high-sensitivity fluorescence time-lapse imaging beyond the shot-noise limit
Source: Nat Biotechnol. 2022 Sep 26;41(2):282–92. doi: 10.1038/s41587-022-01450-8 (PMC9931589; doi:10.1038/s41587-022-01450-8)
Supplement: Supplementary file 2 — Reporting Summary [file 41587_2022_1450_MOESM2_ESM.pdf]

nature research

Corresponding author(s): Qionghai Dai; Lu Fang; Haoqian Wang

Last updated by author(s): Jul 27, 2022

Reporting Summary

Nature Research wishes to improve the reproducibility of the work that we publish. This form provides structure for consistency and transparency in reporting. For further information on Nature Research policies, see our Editorial Policies and the Editorial Policy Checklist.

Statistics

For all statistical analyses, confirm that the following items are present in the figure legend, table legend, main text, or Methods section.

n/a	Confirmed	
☐	☒	The exact sample size (n) for each experimental group/condition, given as a discrete number and unit of measurement
☐	☒	A statement on whether measurements were taken from distinct samples or whether the same sample was measured repeatedly
☐	☒	The statistical test(s) used AND whether they are one- or two-sided<br>*Only common tests should be described solely by name; describe more complex techniques in the Methods section.*
☒	☐	A description of all covariates tested
☒	☐	A description of any assumptions or corrections, such as tests of normality and adjustment for multiple comparisons
☐	☒	A full description of the statistical parameters including central tendency (e.g. means) or other basic estimates (e.g. regression coefficient) AND variation (e.g. standard deviation) or associated estimates of uncertainty (e.g. confidence intervals)
☐	☒	For null hypothesis testing, the test statistic (e.g. F, t, r) with confidence intervals, effect sizes, degrees of freedom and P value noted<br>*Give P values as exact values whenever suitable.*
☒	☐	For Bayesian analysis, information on the choice of priors and Markov chain Monte Carlo settings
☒	☐	For hierarchical and complex designs, identification of the appropriate level for tests and full reporting of outcomes
☐	☒	Estimates of effect sizes (e.g. Cohen's d, Pearson's r), indicating how they were calculated

Our web collection on statistics for biologists contains articles on many of the points above.

Software and code

Policy information about availability of computer code

Data collection	The image acquisition process of our two-photon microscopes was controlled by ScanImage 5.7 (Free release, Vidrio).
Data analysis	Data simulation and result analysis were performed using custom Matlab (R2019b, MathWorks) scripts. All deep learning models reported in this work were implemented with standard libraries of Python (3.6.0), PyTorch (1.8.0, Facebook). Neutrophil segmentation was implemented with the released code of Cellpose v0.6 (https://github.com/MouseLand/cellpose) and StarDist v0.7.3 (https://github.com/stardist/stardist). Manual labeling of neutrophil masks was implemented with the ROI Manager toolbox of Fiji (https://imagej.net/software/fiji/). All results of DeepInterpolation was obtained with the released code (https://github.com/AllenInstitute/deepinterpolation).

For manuscripts utilizing custom algorithms or software that are central to the research but not yet described in published literature, software must be made available to editors and reviewers. We strongly encourage code deposition in a community repository (e.g. GitHub). See the Nature Research guidelines for submitting code & software for further information.

Data

Policy information about availability of data

All manuscripts must include a data availability statement. This statement should provide the following information, where applicable:

- Accession codes, unique identifiers, or web links for publicly available datasets
- A list of figures that have associated raw data
- A description of any restrictions on data availability

We have no restriction on data availability. All source data (including recordings of calcium dynamics, neutrophil migration, and ATP release) have been archived and made publicly available at https://cabooster.github.io/DeepCAD-RT/Datasets/.

Field-specific reporting

Please select the one below that is the best fit for your research. If you are not sure, read the appropriate sections before making your selection.

☒ Life sciences ☐ Behavioural & social sciences ☐ Ecological, evolutionary & environmental sciences

For a reference copy of the document with all sections, see nature.com/documents/nr-reporting-summary-flat.pdf

Life sciences study design

All studies must disclose on these points even when the disclosure is negative.

Sample size	The sample size (n) of each experiment is provided in the figure/table legends in the main manuscript and supplementary information files.
Data exclusions	No data was excluded from the analysis.
Replication	The number of repetitions for each experiment is provided in the figure/table legends in the main manuscript and supplementary information files.
Randomization	Not relevant, as there were no such experimental groups in this study.
Blinding	Not relevant, as there were no such experimental groups in this study.

Reporting for specific materials, systems and methods

We require information from authors about some types of materials, experimental systems and methods used in many studies. Here, indicate whether each material, system or method listed is relevant to your study. If you are not sure if a list item applies to your research, read the appropriate section before selecting a response.

Materials & experimental systems

n/a	Involved in the study	
☐	☒	Antibodies
☒	☐	Eukaryotic cell lines
☒	☐	Palaeontology and archaeology
☐	☒	Animals and other organisms
☒	☐	Human research participants
☒	☐	Clinical data
☒	☐	Dual use research of concern

Methods

n/a	Involved in the study	
☒	☐	ChIP-seq
☒	☐	Flow cytometry
☒	☐	MRI-based neuroimaging

Antibodies

Antibodies used	For neutrophil labeling, we used a commercial antibody: monoclonal anti-Ly-6G/Ly-6C (RB6-8C5), Alexa Fluor 488 (53-5931-82, eBioscience). RRID: AB_469918.
Validation	The antibody is commercial and has been extensively used in previous studies, and our staining pattern was compared to existing literature and images published. The details of these antibodies and their validations can be found in manufacturer's websites: monoclonal mouse Ly-6G/Ly-6C, eBioscience, https://www.thermofisher.com/antibody/product/Ly-6G-Ly-6C-Antibody-clone-RB6-8C5-Monoclonal/53-5931-82

Animals and other organisms

Policy information about studies involving animals; ARRIVE guidelines recommended for reporting animal research

Laboratory animals	For calcium imaging in the mouse brain, we used transgenic mice hybridized between Rasgrf2-2A-dCre mice and Ai148 (TIT2L-GC6f-ICL-tTA2)-D mice expressing Cre-dependent GCaMP6f calcium indicator. For the imaging of neutrophil migration and ATP release, we used C57BL/6J mice labeled by injected dyes or virus. For calcium imaging in the zebrafish brain, we used transgenic zebrafish larvae (Tg(HuC:GCaMP6s)) expressing pan-neuronal GCaMP6s calcium indicator. For calcium imaging in the Drosophila brain, we used transgenic flies crossed between UAS-GCaMP7f and OK107-Gal4 to drive the expression of GCaMP7f in essentially all Kenyon Cells.
Wild animals	none
Field-collected samples	none

Ethics oversight	All experiments involving animals were performed in accordance with the institutional guidelines for animal welfare and have been approved by the Animal Care and Use Committee of Tsinghua University.

Note that full information on the approval of the study protocol must also be provided in the manuscript.
